# EWSR1 associates with the 40S ribosomal subunit and represses translation to maintain endoplasmic reticulum homeostasis

**DOI:** 10.1093/nar/gkag1022

**Published:** 2026-10-29

**Authors:** Zahrat El Oula Hassoun, Loic Ongena, Lionel Condé, Bartimée Galvan, Grégory Fettweis, Michelle Lion, Romuald Soin, Jonathan Bruyr, Florence Lemaitre, Marc Thiry, Emeline Mariavelle, Margaux Claes, Arnaud Blomme, Gerben Menschaert, Jonas Dehairs, Véronique Kruys, Cyril Gueydan, Tina M O’grady, Johannes V Swinnen, Sylvain de Breyne, Théophile Ohlmann, Jean-Claude Twizere, Yvette Habraken, Franck Dequiedt

**Affiliations:** Laboratory of Gene Expression and Cancer, GIGA Institute, University of Liège, 4000 Liege, Belgium; Laboratory of Gene Expression and Cancer, GIGA Institute, University of Liège, 4000 Liege, Belgium; CIRI, Centre International de Recherche en Infectiologie, Univ Lyon, INSERM U1111, Université Claude Bernard Lyon 1, CNRS UMR5308, ENS de Lyon, F-69007 Lyon, France; Laboratory of Gene Expression and Cancer, GIGA Institute, University of Liège, 4000 Liege, Belgium; Laboratory of Gene Expression and Cancer, GIGA Institute, University of Liège, 4000 Liege, Belgium; Laboratory of Gene Expression and Cancer, GIGA Institute, University of Liège, 4000 Liege, Belgium; Laboratoire de Biologie Moléculaire du Gène, Institut de Biologie et de Médecine Moléculaires, Université Libre de Bruxelles (ULB), 6041 Gosselies, Belgium; Laboratory of Gene Expression and Cancer, GIGA Institute, University of Liège, 4000 Liege, Belgium; Laboratory of Gene Expression and Cancer, GIGA Institute, University of Liège, 4000 Liege, Belgium; Laboratory of Cell and Tissue Biology, GIGA Institute, University of Liège, 4000 Liege, Belgium; Laboratory of Gene Expression and Cancer, GIGA Institute, University of Liège, 4000 Liege, Belgium; Laboratory of Gene Expression and Cancer, GIGA Institute, University of Liège, 4000 Liege, Belgium; Laboratory of Metabolic Regulation, GIGA Institute, University of Liège, 4000 Liege, Belgium; Biobix, Laboratory of Bioinformatics and Computational Genomics, Ghent University, 9000 Ghent, Belgium; Laboratory of Lipid Metabolism and Cancer, Department of Oncology, Leuven Cancer Institute and Leuven Institute for Single Cell Omics (LISCO), KU Leuven, Leuven B-3000, Belgium; Laboratoire de Biologie Moléculaire du Gène, Institut de Biologie et de Médecine Moléculaires, Université Libre de Bruxelles (ULB), 6041 Gosselies, Belgium; Laboratoire de Biologie Moléculaire du Gène, Institut de Biologie et de Médecine Moléculaires, Université Libre de Bruxelles (ULB), 6041 Gosselies, Belgium; Laboratory of Gene Expression and Cancer, GIGA Institute, University of Liège, 4000 Liege, Belgium; Laboratory of Lipid Metabolism and Cancer, Department of Oncology, Leuven Cancer Institute and Leuven Institute for Single Cell Omics (LISCO), KU Leuven, Leuven B-3000, Belgium; CIRI, Centre International de Recherche en Infectiologie, Univ Lyon, INSERM U1111, Université Claude Bernard Lyon 1, CNRS UMR5308, ENS de Lyon, F-69007 Lyon, France; CIRI, Centre International de Recherche en Infectiologie, Univ Lyon, INSERM U1111, Université Claude Bernard Lyon 1, CNRS UMR5308, ENS de Lyon, F-69007 Lyon, France; Laboratory of Viral Interactomes Networks, GIGA Institute, University of Liège, 4000 Liege, Belgium; Laboratory of Gene Expression and Cancer, GIGA Institute, University of Liège, 4000 Liege, Belgium; Laboratory of Gene Expression and Cancer, GIGA Institute, University of Liège, 4000 Liege, Belgium

## Abstract

FET family proteins (FUS, EWSR1, and TAF15) have garnered significant attention due to their involvement in oncogenic fusions and mutations in various cancers and neurodegenerative disorders. Although these highly conserved RNA-binding proteins are widely described as multifunctional regulators of gene expression, their precise molecular functions remain incompletely understood. Here, we uncover a previously unrecognized role for EWSR1 as a selective translational repressor in cancer cells. EWSR1 specifically targets mRNAs involved in lipid metabolism and endoplasmic reticulum (ER) homeostasis. Loss of EWSR1 disrupts ER morphology and impairs activation of the unfolded protein response of the ER (UPR^ER^). Mechanistically, EWSR1 represses translation upon association with target mRNAs, and its conserved region spanning two arginine–glycine–glycine (RGG) boxes contributes to association with the 40S ribosomal subunit. These findings define a novel role for EWSR1 in post-transcriptional gene regulation and suggest that perturbations in its translational activity may underlie the pathogenesis of FET-associated diseases.

## Graphical Abstract

**Graphical Abstract:**
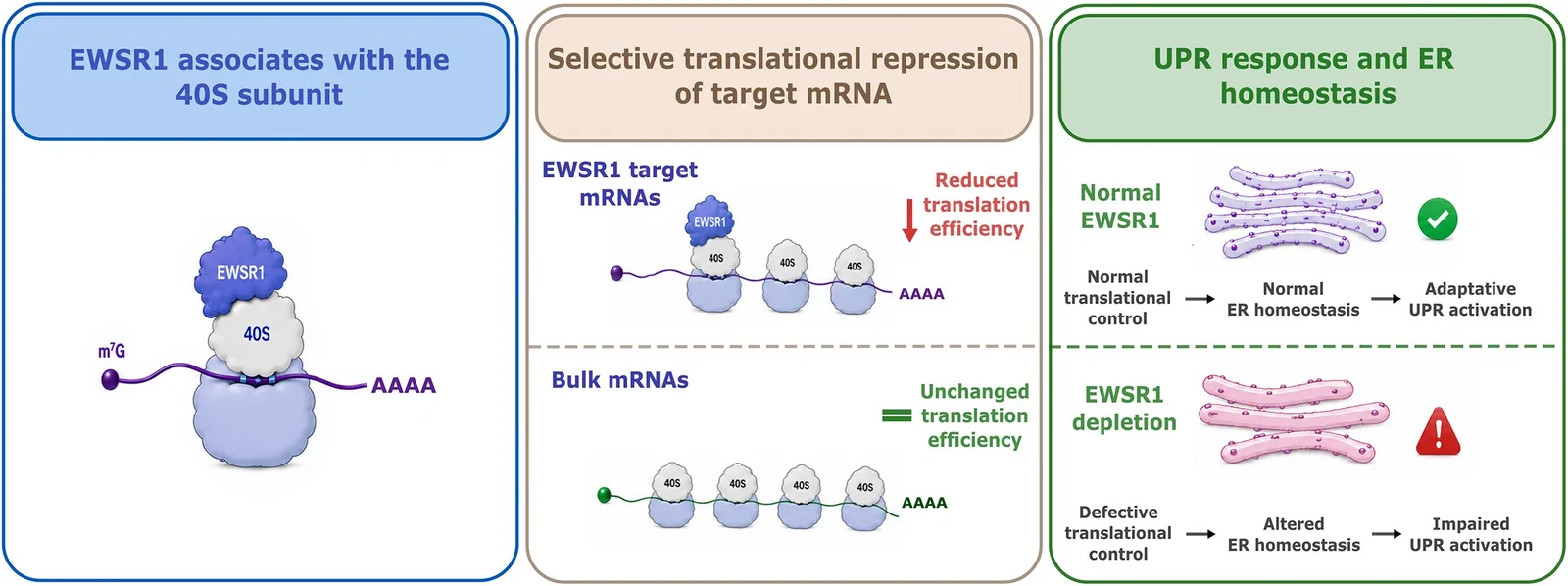

## Introduction

While transcriptional control represents a fundamental layer of gene regulation, post-transcriptional mechanisms, including cytoplasmic processes such as mRNA transport, degradation and translation, introduce additional complexity, and play a crucial role in fine-tuning protein synthesis. These post-transcriptional processes are orchestrated by RNA-binding proteins (RBPs), which interact with mRNAs to form messenger ribonucleoprotein complexes (mRNPs) [1]. RBPs often regulate more than one aspect of RNA metabolism [2]. A notable example is the FET family of RBPs, including FUS (Fused in sarcoma), EWSR1 (Ewing sarcoma breakpoint region 1), and TAF15 (TATA-box binding protein associated factor 15). These proteins share high structural and functional similarities and are ubiquitously expressed in human cells, ranking among the most abundant proteins, underlying their roles in diverse cellular processes [3]. A defining feature of FET proteins is their N-terminal prion-like domain (PrLD), which provides phase separation properties [4]. This PrLD functions as a potent transactivation domain with oncogenic potential, as evidenced by its frequent fusion with the DNA-binding domains of various transcription factors in sarcomas and leukemias [5]. In contrast, the C-terminal region of the FET protein is almost systematically lost in these oncogenic fusions, suggesting that this domain may have an antitumor function. The C-terminal region of FET proteins harbors multiple nucleic acid-binding domains, including three arginine–glycine–glycine (RGG) boxes [6], an RNA recognition motif (RRM) [7] and a zinc finger (ZnF) domain [3]. Among the three RGG boxes, RGG2 and RGG3 are the most conserved across all FET members, whereas TAF15 RGG1 is highly divergent and embedded within the PrLD [8]. The conserved RGG2–ZnF–RGG3 region contributes to nucleic acid binding of FET proteins to specific RNA and DNA structures, including RNA stem loops, RNA G-quadruplexes (rG4), and RNA:DNA hybrids [9–11, 12]. Beyond nucleic acid interactions, FET RGG boxes are also implicated in protein–protein interactions [13, 14, 15]. The ability of FET proteins to engage in broad yet specific molecular interactions with proteins and nucleic acids via their RGG boxes may be particularly relevant to their physiological functions. Notably, mutations in the RGG2–ZnF–RGG3 region of FET members are frequently observed in neurological disorders such as amyotrophic lateral sclerosis (ALS), further highlighting its functional significance [16].

Despite their high pathological relevance in multiple neurodegenerative disorders and cancers, the normal molecular functions of FET proteins remain poorly understood. FET members are predominantly found in the nucleus, where they associate with chromatin, the RNA Pol II complex and various transcription factors affecting transcription at different levels [3, 17, 18]. Several lines of evidence also suggest the involvement of FET proteins in nuclear mRNA processing and in particular pre-mRNA splicing [19–21, 22] and mRNA export [23], regulation of the DNA damage repair [24, 25], and pri-miRNA processing [26].

FET members are also found in the cytoplasm [27]. FUS is a component of neuronal RNA transport granules that are involved in localized translation of specific mRNA [28]. In stress conditions, FET proteins accumulate in stress granules (SGs), which contain stalled translation preinitiation complexes [29]. These observations suggest that FET proteins might control mRNA fate in the cytosol, particularly its translation. The potential role of FET proteins in translation remains essentially unexplored and the limited supporting evidence has been derived exclusively from studies on FUS, with conflicting results [30, 31, 32]. A role of FUS in translation could be highly relevant for FUS-proteinopathies such as ALS, frontotemporal lobar degeneration (FTLD) or other neurodegenerative disorders [33]. Beyond the above findings on FUS, it is unclear whether other members of the FET family contribute to translation regulation. Moreover, the molecular mechanisms by which FET proteins impact translation have yet to be elucidated. In this study, we demonstrate that EWSR1 represses translation of specific mRNAs linked to ER homeostasis. Mechanistically, we show that translational inhibition by EWSR1 requires its association with target transcripts, while its RGG2–ZnF–RGG3 region contributes both to translational repression and to association with the 40S ribosomal subunit. Our results identify a new function for EWSR1, which may unravel novel therapeutic opportunities for diseases associated with mutations affecting its RGG2–ZnF–RGG3 region, including cancers, such as Ewing sarcomas and other soft tissue neoplasms or neurological disorders such as ALS [34, 35].

## Materials and methods

### Antibodies and reagents

All antibodies used in this study are listed in the Table 1 . Chemical and reagents used in this study are listed in Table 2.

**Table 1.** Antibodies used in this study with the provider and host species

Antibodies	Source	Host species
EWSR1	Santa Cruz Biotechnologies, Inc (sc-398 318)	Mouse
EWSR1	Cell Signaling Technology (#11910)	Rabbit
FLAG	Merck (F3165)	Mouse
FLAG	Merck (F7425)	Rabbit
GAPDH	Santa Cruz Biotechnologies, Inc. (sc-166545)	Mouse
β-tubulin	Abcam (ab6046)	Rabbit
Histone H3	Santa Cruz Biotechnologies (sc-8654)	Goat
Hsp90	Cell Signaling Technology (#4877)	Rabbit
Puromycin	Merck (MABE343-clone 12D10)	Mouse
RPS6	Cell Signaling Technology (#2217)	Rabbit
RPS23	Santa Cruz Biotechnologies (sc-100837)	Mouse
RPL26	Bethyl Laboratories, Inc. (A-300-686)	Rabbit
RPL22	Santa Cruz Biotechnologies (sc-373993)	Mouse
RPS5	Santa Cruz Biotechnologies (sc-390935)	Mouse
eIF2α	Cell Signaling Technology (#9722)	Rabbit
p-eIF2α	Cell Signaling Technology (#9721)	Rabbit
PERK	Santa Cruz Biotechnologies (sc-377400)	Mouse
ATF4	Cell Signaling Technology (#11815)	Rabbit
ATF6	Cell Signaling Technology (#65880)	Rabbit
CHOP	Cell Signaling Technology (#2895)	Mouse
p21	Santa Cruz Biotechnologies (sc-397)	Rabbit
IRE1α	Santa Cruz Biotechnologies (sc-390960)	Mouse
p-IRE1α (phospho S724)	Abcam (ab124945)	Rabbit
XBP1s	Cell Signaling Technology (#12782)	Rabbit
eIF3η	Santa Cruz Biotechnologies (sc-137214)	Mouse
RBPMS	Proteintech (15187-1-AP)	Rabbit
RPL4	Santa Cruz Biotechnologies (sc-100838)	Mouse
RACK1	Santa Cruz Biotechnologies (sc-17754)	Mouse
eIF4E	Cell Signaling Technology (#9742)	Rabbit
eIF5	Santa Cruz Biotechnologies (sc-28309)	Mouse
eIF4G	Santa Cruz Biotechnologies (sc-133155)	Mouse
GST	Santa Cruz Biotechnologies (sc-138)	Mouse
Mouse IgG	Santa Cruz Biotechnologies (sc-2025)	Mouse
Rabbit IgG	Cell Signaling Technolgy (#2729)	Rabbit

**Table 2.** Chemicals and reagents used in this work with their source and identifier

Chemicals and reagents	Source	Identifier
Spectinomycin	Merck	S4014
Puromycin	InvivoGen	ant-pr-1
Ampicillin	Merck	A0166
Thapsigargin	Merck	T9033
Palmitic acid	Merck	P0500-10G
Tunicamycin	Merck	T7765
Cycloheximide	Merck	C4859
Polyethyleneimine (PEI)	Merck	919012
Luminol, sodium salt	Merck	A4685
*p*-Coumaric acid	Merck	C9008
RNAse A	Thermo Fisher Scientific, Inc.	EN0531
RNAse T1	Thermo Fisher Scientific, Inc.	EN0541
Proteinase K	Merck	P2308
JetPrime^®^ DNA and siRNA transfection reagent	Avantor	PPLU101000027
LipofectamineTM 2000 Transfection reagent	Thermo Fisher Scientific, Inc.	11668019
cOmplete Protease Inhibitors	Merck	4693132001
Halt^™^ Phosphatase Inhibitors	Thermo Fisher Scientific, Inc.	78440
Protector RNase Inhibitor	Merck	3335399001
Anti-FLAG M2 agarose beads	Merck	A2220
m7GTP-Agarose	Creative BioMart	m7GTP-001A
Oligo d(T)_25_ magnetic beads	New England BioLabs	S1419S
PureProteome Protein A/G Mix Magnetic Beads	Merck	LSKMAGAG02
TakyonTM No ROX SYBR 2X MasterMix blue dTTP	Kaneka Eurogentec S.A.	UF-NSMT-B0701
Twinlite Firely and Renilla Luciferase Reporter Gene Assay System	Revvity	6066709
NucleoSpin Plasmid EasyPure	Macherey-Nagel	740727.50
Nucleospin RNA kit	Macherey-Nagel	740955.50
TRIzol^™^ reagent	Thermo Fischer Scientific, Inc.	15596018

### Cell lines, culture conditions, transfections, and treatments

HeLa, HEK293, A2780A, U2OS, and MCF-7 cells were purchased from American Type Culture Collection (ATCC). All cells were cultured in a 37°C, 5% CO_2_ humidified incubator. All cell lines were maintained in DMEM high glucose (Biowest) with 10% FBS (Thermo Fisher Scientific, Inc.) and 1% Penn/Strep (Biowest) and were routinely checked for mycoplasma by the GIGA-Viral vectors facility using MycoAlert^™^ Mycoplasma Detection Kit (Lonza). Culture medium was changed every 2 days and cell confluency was usually maintained <80%, unless specified otherwise.

For MS2-tethering assays and co-immunoprecipitation experiments, cells were transfected with suitable DNA constructs using polyethyleneimine (PEI) with a 1:2 DNA/PEI ratio. For transient EWSR1 knockdown experiments, the following siRNAs, previously used in [36] were purchased from Eurogentec (Belgium): *siEWSR1#1*, 5′-GAUGAAGACUCUGACAACAtt-3′; *siEWSR1#2*, 5′- GAUUAUUCCUUGUCUGUACUUtt-3′, and control *siRNA* (*siCTL*, SR-CL000-005). siRNAs were transfected with JetPrime (Avantor) or Lipofectamine RNAi MAx (Thermo Fischer Scientific, Inc.), according to the manufacturer’s instructions. Cells were processed for downstream analyses 48 h after DNA or siRNA transfection, unless specified otherwise.

When indicated, cells were treated with tunicamycin (diluted in DMSO and used at 0.5 μg/ml for 5 h), thapsigargin (diluted in DMSO and used at 500 nM for 24 h), and palmitic acid (diluted in DMEM/10% BSA and used at 800 μM for 24 h) 48 h after transfection with *siCTL* or *siEWSR1#1* or *siEWSR1#2*. After treatment, cells were lysed in Laemmli buffer and lysates were sonicated, boiled for 5 min at 100°C and analyzed by SDS–PAGE and western blotting.

### Plasmids and cloning

Open reading frame (ORF) encoding human EWSR1 was obtained as *pDONR223* from the human ORFeome v8.1 (The Center for Cancer Systems Biology, Dana-Farber Cancer Institute, CCSB-DFCI). N-terminal domain of EWSR1 (NTD), C-terminal domain of EWSR1 (CTD), CTD mutants, and EWSR1 mutant were inserted into *pDONR223* by BP cloning (Gateway Recombination Technology, Thermo Fischer Scientific, Inc.) with specific primers flanked at the 5′ site by the following AttB1 and AttB2 Gateway sites: 5′-GGGGACAACTTTGTACAAAAAAGTTGGC(ATG)-3′ (AttB1) and 5′-GGGGACAACTTTGTACAAGAAAGTTGA-3′ (AttB2). The ΔRGG2, ΔZnF, ΔRGG3, ΔRGG2-ZnF, ΔZnF-RGG3, ΔRGG2-ZnF-RGG3 CTD mutants, and the EWSR1 ΔRGG2-ZnF-RGG3 mutant were generated from EWSR1 CTD or EWSR1 using the overlap extension PCR cloning procedure and cloned into *pDONR223*. Inserts from *pDONR223* were subsequently transferred by LR cloning (Thermo Fischer Scientific, Inc.) into different destination vectors: *pDEST1899* (FLAG N-terminal tag), *pGEX-6P-1* (Addgene). For the MS2-tethering assay, *pDEST1899* Flag-tagged ORFs were subcloned in the *pN-MS2-CP* (MS2 N-terminal tag) as described before [37]. The bidirectional reporters encoding a control Firefly luciferase (F-Luc) and a targeted Renilla luciferase carrying or lacking 4 or 8 repeats of the binding sequence for the MS2 coating peptide in its 3′UTR (*pCMV-RLuc-0MS2, pCMV-RLuc-4MS2*, and *pCMV-RLuc-8MS2*, respectively) have been described elsewhere [37]. To generate the *CrPV- RLuc-0MS2* and *CrPV-RLuc-8MS2* reporters, the CrPV IRES sequence was amplified from CrPV-luciferase-MS2 reporter [a gift from Dr. Nicola K. Gray (MRC Human Genetics Unit, United Kingdom)] using the following primers: 5′-GGTGGCGGCTAGCAAAGCAAAAATGTGATCTTGC-3′ (Forward) and 5′- GGTGGCGGCTAGCCTATCTTGAAATGTAGCAGGTAA-3′ (Reverse). To generate *PV-RLuc-0MS2* or *PV-RLuc-8MS2*, the PV IRES sequence was amplified from PV-luciferase-MS2 reporter [a gift from Dr. Nicola K. Gray (MRC Human Genetics Unit, United Kingdom)] using the following primers: 5′-GGTGGCGGCTAGCGGGAACAAAAGCTGGTACCG-3′ (Forward) and 5′-GGTGGCGGCTAGCCGAGCTGAATCTCTATAATAATTAATGG-3′ (Reverse). The IRES PCR products were digested with NheI and ligated into the NheI site of *pCMV-RLuc-0MS2* and *pCMV-RLuc-8MS2*, respectively. All constructs were verified by Sanger sequencing at the GIGA-Genomics facility (University of Liège). All constructs used in this study are listed in Table 3. Cloning and sequencing primers are listed in Table 4.

**Table 3.** Plasmids used in this work with their source and identifier

Plasmids	Source
Human ORFeome v7.1 and v8.1	The Center for Cancer Systems Biology (CCSB- DFCI) http://horfdb.dfci.harvard.edu/
*pDONR223*	[38]
*pDEST1899*	[37]
*pN-MS2-CP*	[37]
*RLuc*-4MS2	[37]
*RLuc-*8MS2	[37]
*RLuc*-0MS2	[37]
*pGL3-promoter*	Addgene
*pGEX-4T1*	Addgene
*pGEX-6p-1*	Addgene
*TISU-RLuc-6xMS2-poly(A)*	[39]

**Table 4.** Cloning primers used in this work

Target (Cloning)	Forward	Reverse
RGG1-RRM	GGGGACAACTTTGTACAAAAAAGTTGGCATGGGAGGATTTTCCGGACCAGG	GGGGACAACTTTGTACAAGAAAGTTGAAAGGGAGACTTTAAGTTTGC
ΔRGG1-RRM	GGGGACAACTTTGTACAAAAAAGTTGGCATGCGGGGTGGTCTGCCACCCCG	GGGGACAACTTTGTACAAGAAAGTTGAGTAGGGCCGATCTCTGCG
ΔNLS	GGGGACAACTTTGTACAAAAAAGTTGGCATGGGAGGATTTTCCGGACCAGG	GGGGACAACTTTGTACAAGAAAGTTGATCCTCCACGTCCTCCTCTTCTTCC
ΔRGG3	GGGGACAACTTTGTACAAAAAAGTTGGCATGGGAGGATTTTCCGGACCAGG	GCCTTTATCCATTTTTCCAGGAGGCTTTGGGGCCTTACACTG
	CAGTGTAAGGCCCCAAAGCCTCCTGGAAAAATGGATAAAGGC	GGGGACAACTTTGTACAAGAAAGTTGAGTAGGGCCGATCTCTGCG
ΔZnF	GGGGACAACTTTGTACAAAAAAGTTGGCATGGGAGGATTTTCCGGACCAGG	GGGTGGCGGGAGGAAGCCTTCGTGCTGGACGTTTCCTCCTCC
	GGAGGAGGAAACGTCCAGCACGAAGGCTTCCTCCCGCCACCC	GGGGACAACTTTGTACAAGAAAGTTGAGTAGGGCCGATCTCTGCG
ΔZnF-RGG3	GGGGACAACTTTGTACAAAAAAGTTGGCATGGGAGGATTTTCCGGACCAGG	GCCTTTATCCATTTTTCCAGGGTGCTGGACGTTTCCTCCTCC
	GGAGGAGGAAACGTCCAGCACCCTGGAAAAATGGATAAAGGC	GGGGACAACTTTGTACAAGAAAGTTGAGTAGGGCCGATCTCTGCG
ΔRGG2	GGGGACAACTTTGTACAAAAAAGTTGGCATGGGAGGATTTTCCGGACCAGG	GGGACACTGCCAGTCTCCAGCTCGACTGTTCATTGGAGGCTTCTTCCG
	CGGAAGAAGCCTCCAATGAACAGTCGAGCTGGAGACTGGCAGTGTCCC	GGGGACAACTTTGTACAAGAAAGTTGAGTAGGGCCGATCTCTGCG
ΔRGG2-ZnF	GGGGACAACTTTGTACAAAAAAGTTGGCATGGGAGGATTTTCCGGACCAGG	GGGTGGCGGGAGGAAGCCTTCACTGTTCATTGGAGGCTTCTTCCG
	CGGAAGAAGCCTCCAATGAACAGTGAAGGCTTCCTCCCGCCACCC	GGGGACAACTTTGTACAAGAAAGTTGAGTAGGGCCGATCTCTGCG
ΔRGG2-ZnF-RGG3	GGGGACAACTTTGTACAAAAAAGTTGGCATGGGAGGATTTTCCGGACCAGG	GCCTTTATCCATTTTTCCAGGACTGTTCATTGGAGGCTTCTTCCG
	GGGGACAACTTTGTACAAAAAAGTTGGCATGGCGTCCACGGATTACAGTACC	GGGGACAACTTTGTACAAGAAAGTTGAGTAGGGCCGATCTCTGCG
EWSR1 ΔRGG2-ZnF-RGG3	GGGGACAACTTTGTACAAAAAAGTTGGCATGGCGTCCACGGATTACAGTACC	GCCTTTATCCATTTTTCCAGGACTGTTCATTGGAGGCTTCTTCCG
	CGGAAGAAGCCTCCAATGAACAGTCCTGGAAAAATGGATAAAGGC	GGGGACAACTTTGTACAAGAAAGTTGAGTAGGGCCGATCTCTGCG

### RNA isolation and quantitative PCR

For MS2-tethering assays and RT-PCR of unspliced (u) and spliced (s) XBP1, total RNA was isolated using the Nucleospin RNA kit (Macherey-Nagel). For RNA immunoprecipitations, total RNA was purified with TRIzol^™^ Reagent and reverse-transcribed with random primers using the RevertAid H Minus First Strand cDNA Synthesis Kit (Thermo Fisher Scientific, Inc). cDNA was amplified and quantified in triplicate using FastStart SYBR Green Master mix on a LightCycler 480 instrument (Merck). In MS2-tethering studies, relative levels of *RLuc* mRNA were assessed using primers specific for *FLuc* [5′- TGAGAACTTCAGGCTCCTGG-3′ (Forward) and 5′-GCCTTATGCAGTTGCTCTCC-3′ (Reverse)] and *RLuc* [5′-TCTTTTTCGCAACGGGTTT-3′ (Forward) and 5′- GCCCAGTTTCTATTGGTCTCC-3′ (Reverse)]. Fold changes were calculated relatively to the MS2-CP empty vector condition with the ΔΔ*C*t method. Primers used for RT-qPCR of unspliced (u) and spliced (s) XBP1 were 5′-CTGGAACAGCAAGTGGTAGA-3′ (Forward) and 5′- CTGGGTCCTTCTGGGTAG-3′ (Reverse) as described in [40].

### Western blotting

Cells were washed with cold PBS, directly lysed in Laemmli buffer, sonicated, and boiled for 5 min at 100°C. Protein extracts were separated by sodium dodecyl sulfate-polyacrylamide gel (SDS–PAGE) electrophoresis and transferred onto nitrocellulose membrane. Membranes were blocked for 1 h with 5% non-fat milk and incubated in 4% BSA overnight at 4°C with primary antibodies followed by HRP-conjugated secondary antibodies. Proteins were detected by chemiluminescence using a homemade revelation solution obtained by mixing Solution A [50 mg of Luminol sodium salt (Merck, A4685) in 200 ml 0.1 M Tris–HCl (pH 8.6)], Solution B [11 mg *p*-Coumaric acid (Merck, C9008) in 10 ml DMSO], and Solution C (35% H_2_O_2_) in a 1:0.1:0.0003 ratio. Images were acquired with ImageQuant LAS 4000 device (GE Healthcare, Chicago, USA) or Amersham ImageQuant 800 (Cytivia, Washington, USA), and quantified using ImageJ. Primary antibodies are listed in Table 1.

### MS2-tethering assay

Cells were transfected in six-well plates format with control MS2-CP empty vector or indicated MS2-CP-tagged constructs, together with bidirectional reporter constructs encoding a firefly (FLuc) and Renilla (RLuc) luciferase genes, the latter lacking or carrying 4 or 8 repeats of the binding sequence for the MS2 coating peptide in its 3′UTR (*RLuc-0MS2, RLuc-4MS2*, and *RLuc-8MS2*, respectively). For CrPV- or PV-RLuc reporters-tethering assays, cells were transfected with control MS2-CP or various MS2-CP-tagged constructs, together with a *CrPV-* or *PV-RLuc* bidirectional reporter encoding a control Firefly luciferase (FLuc) and a reporter Renilla luciferase (RLuc) genes, the latter carrying or lacking eight repeats of the binding sequence for the MS2 coating peptide in its 3′UTR (*CrPV/PV-RLuc-8* and *CrPV/PV- RLuc-0*, respectively). For *TISU-RLuc-6xMS2-poly(A)* or *RLuc-6xMS2-poly(A)* reporters- tethering assays, cells were transfected with control MS2-CP empty vector or various MS2-CP-tagged constructs, together with *TISU-RLuc-6xMS2-poly(A)* or *RLuc-6xMS2-poly(A)* reporters and *pGL3-Firefly* vector (Addgene) (a control Firefly luciferase vector). For measuring luciferase activities in all these assays, cell lysis and luciferase activity were performed in triplicate on a TriStar2 S LB 942 luminometer (Berthold Technologies Gmbh & Co.KG, Bad Wildbad) with Twinlite Firefly and Renilla Luciferase Reporter Gene Assay System (Revvity).

### SUnSET assay

HeLa cells were transfected with *siCTL* or *siEWSR1#1* and 48 h post-transfection, cells were incubated with 1μM puromycin for 30 min, washed 2 x with PBS before lysis in Laemmli buffer. Lysates were sonicated, boiled and subsequently analyzed by SDS–PAGE and western blotting.

### Proliferation assay

Forty-eight hours post-KD of EWSR1 with *siEWSR1#1* or *siEWSR1#2*, HeLa cells were seeded at a density of 7000 cells per well of a 24-well plate. Cell growth was assessed by monitoring cell confluence using time-lapse microscopy (IncuCyte S3, Essen Bioscience, Sartorius) every 4 for 72 h, in triplicate.

### Cell cycle analysis

Cells were trypsinized, rinsed three times with cold PBS, fixed with ice-cold 70% ethanol for 30 min at −20°C, and used immediately or stored at −20°C. Fixed cells were permeabilized with PBS with 0.25% Triton X-100 on ice for 5 min. Cells were then treated with RNase A (200 μg/ml) for 30 min at 37°C. Propidium iodide was added (40 μg/ml), and cells were immediately subjected to FACS analysis on a FACSCalibur flow cytometer (BD Biosciences, San Jose, CA, USA). Cell cycle distribution analysis was performed using BD CellQuest^™^ Pro Analysis software (BD Biosciences, San Jose, CA, USA). A minimum of three independent experiments were performed.

### Co-immunoprecipitation (co-IP)

HeLa cells co-expressing tagged interaction partners were lysed in IPLS (ImmunoPrecipitation Low Salt; Tris–HCl, pH 7.5, 50 mM, EDTA, pH 8, 0.5 mM, 0.5% NP-40, 10% glycerol, and 120 mM NaCl) with 1× cOmplete Protease Inhibitor and 1× Halt^™^ Phosphatase Inhibitors for 30 min at 4°C and cleared by centrifugation. When specified, cleared lysates were incubated at 37°C for 30 min with or without 10 μg/ml RNase A (Thermo Fisher Scientific, Inc.) or 2000 U RNase T1 (Thermo Fisher Scientific, Inc.). Lysates were then incubated with anti-FLAG M2 agarose beads (Merck) for 2 h at 4°C. Beads were washed four times with IPLS and immunoprecipitates were finally boiled in SDS loading buffer and analyzed by SDS–PAGE and western blotting. For endogenous co-immunoprecipitations, HEK293 cell lysates were prepared in IPLS, precleared with Protein A/G magnetic beads for 1 h at 4°C, and incubated overnight with 1.5 μg of relevant antibody or control mouse or rabbit IgG. Then, samples were incubated with Protein A/G magnetic beads for 2 h at 4°C. Beads were washed four times with IPLS buffer, boiled in 2× SDS loading buffer and quickly centrifuged at high speed. Supernatants were analyzed by SDS–PAGE and western blotting.

### Oligo(dT) pulldowns

HeLa cells cultured in 15 cm dishes were washed twice in cold PBS and UV cross-linked (0.4 J/cm^2^ of 365-nm UV light with a Stratalinker 2400). UV-cross-linked cells were divided into two fractions: total and cytoplasmic extracts. For total extract, cells were lysed in oligo(dT) lysis buffer (Tris–HCl, pH 7.5, 20 mM, 500 mM LiCl, 0.5% SDS, 1 mM EDTA, 1 mM DTT, cOmplete Protease Inhibitor, Halt^™^ Phosphatase Inhibitors, and Protector RNase Inhibitor), then cell lysates were homogenized by four passages through a syringe with a 0.4-mm diameter needle. For cytoplasmic extract fraction, cells were resuspended in cytoplasmic extraction buffer (Tris–HCl, pH 7.5, 20 mM, 100 mM KCl, 5 mM MgCl_2_, 0.3% Nonidet *P*-40, 1 mM DTT, cOmplete Protease Inhibitor, Halt^™^ Phosphatase Inhibitors, and Protector RNase Inhibitor). After centrifugation, the supernatant was collected and lysed in 2× lysis buffer (Tris–HCl, pH 7.5, 20 mM, 0.9 mM LiCl, 0.7% SDS, 2 mM EDTA, 1 mM DTT, cOmplete Protease Inhibitor, Halt^™^ Phosphatase Inhibitors, and Protector RNase Inhibitor). Cleared total and cytoplasmic fractions were then incubated at 37°C for 30 min with or without RNase A [neither DTT nor RNase protector was added in the RNase-treated samples used as negative controls for oligo(dT) pulldown]. One percent of each cell lysate was kept for input, and the rest was incubated with oligo d(T)_25_ magnetic beads for 2 h at 4°C (25 μl of beads per 15-cm culture dish). Pulldowns were washed 1× with wash buffer I (Tris–HCl, pH 7.5, 20 mM, 500 mM LiCl, 0.1% SDS, 1 mM EDTA, and 1 mM DTT), 1× with wash buffer II (Tris–HCl, pH 7.5, 20 mM, 500 mM LiCl, 1 mM EDTA, and 1 mM DTT) and 1× with wash buffer III (Tris–HCl, pH 7.5, 20 mM, 200 mM LiCl, 1 mM EDTA, and 1 mM DTT). Beads and inputs were resuspended in 2× SDS loading buffer, sonicated for 5 min to reduce viscosity, boiled, and analyzed by SDS–PAGE and western blotting.

### RNA immunoprecipitation-qPCR analysis

Fifty microliters of Protein A/G magnetic beads were washed twice in 1 ml of PBS 0.02% Tween and incubated with 10 µg anti-EWSR1 (sc-398318, Santa Cruz) antibody or control mouse IgG (Santa Cruz) at 4°C overnight with rotation in 500 µl of PBS 0.02% Tween. Cells (2 × 15 cm plates) were washed 2× with ice-cold PBS, scratched off the plates, pelleted by centrifugation (4°C, 5 min, 200 × *g*), and lysed in 2 ml RIPA (Tris–HCl, pH 8.0, 50 mM, 150 mM NaCl, 1.0% IGEPAL CA-630, 0.5% sodium deoxycholate, and 0.1% SDS) with 1× cOmplete Protease Inhibitor during a 30 min incubation at 4°C with rotation. Cell lysates were then sonicated on ice and cleared by centrifugation for 10 min at 4°C at 9300 × *g*. Proteins were quantified with Pierce BCA protein assay kit (Thermo Fisher Scientific, Inc.). An aliquot was used for RNA input and was treated with proteinase K before RNA extraction with TRIzol^™^ Reagent. For immunoprecipitation, lysates (400 μg) were incubated with antibody-loaded Protein A/G magnetic beads overnight with rotation at 4°C. Supernatant was removed and beads were washed 5 times with 1 ml RIPA buffer. RNA–protein complexes were then eluted by incubation with 100 μl of elution buffer (Tris–HCl, pH 8, 100 mM, 10 mM Na_2_-EDTA, and 1% SDS in H_2_O) for 3 min at 90°C. Proteins were digested with proteinase K treatment (200 µg/ml, 1 h at 37°C and 45 min at 70°C) and RNA from input or beads were extracted with 1 ml TRIzol^™^ Reagent for RT-qPCR analysis. GAPDH mRNA was used as control. All primers used for RIP-qPCR experiments are listed in Table 5.

**Table 5.** RIP-qPCR primers used in this work

Target gene	Forward	Reverse	Ref.
*FADS1*	CAGCTTTGAGCCCACCAAGAA	AGCAAGATGTGCAGCAGGTACAG	[41]
*FADS2*	TCATGACCATGATCGTCCATAAGAA	GCTCCCAGGATGCCG TAGAA	[41]
*ELOVL5*	TGAGGCAGTGGTCAAACAGGTA	AGATATGTCATGAGTGGTTCCAAGA	[41]
*ELOVL6*	GTGGTCGGCACCTAATG	AACCCTGGTCACAAACTG	[42]
*LPCAT1*	CGACTGAGCGCCCTGCAGAA	AAGGGCCAGGCCAGCAGCAT	[43]
*LAMP1*	AATGTCTGCAGCTCAAGGGC	CAGGATCACCCCGAATGTCA	NA
*SLC39A1*	CTGGCTGTGGGATCCTCTTC	CCATGCCCTCTAGCACAGAC	NA
*HSD17B12*	GGTCTTGAAATCGGCATCTTAGT	ATGTTCAGAATAGCCCCTTTGG	[44]
SEC61G	AAAGGACTCCATTCGGCTGGTT	CAAAGAAGCCAATGAATCCC	[45]
*KDELR1*	CTTCTACTCTGTGGCCCCAAT	AAAGATCTTGGACCCTGCCC	NA
*ZNF664*	AAAGCCACAATCATTGCCCG	GAGCTCCTCAAGGGGGAGTA	NA
*RPL32*	CATCTCCTTCTCGGCATCA	AACCCTGTTGTCAATGCCTC	NA
*GAPDH*	TTGCCATCAATGACCCCTTCA	CGCCCCACTTGATTTTGGA	NA
*18S*	AACCCGTTGAACCCCATT	CCATCCAATCGGTAGTAGCG	[37]

NA: Not available/applicable. Ref: reference.

### Polysome profiling

Cells were treated with Cycloheximide (100 μg/ml) for 5 min to fix polysomes, and harvested in buffer containing 25 mM Hepes, 100 mM KCl, 5 mM MgCl_2_, 0.5% Nonidet P-40, 2 μg/ml heparin, and 10 μg/ml cycloheximide. Cell extracts were cleared by centrifugation at 13 000 × rpm for 10 min at 4°C and loaded onto a 15%–50% linear sucrose gradient. Samples were ultracentrifuged for 2 h at 39 000 × rpm in a SW41 rotor and subsequently fractionated with a gradient fractionator (Brandel). Absorbance was read at 260 nm and collected fractions were stored at −80°C for subsequent analysis by SDS–PAGE and western blotting. Polysomes profiles were compared and quantified with QuAPPro R shiny app.

### Nuclear and cytoplasmic fractionation

HeLa cells were trypsinized, washed twice with cold PBS, and lysed in a volume of CLB buffer (cytoplasmic lysis buffer; Tris–HCl, pH 7.9, 10 mM, 340 mM sucrose, 3 mM CaCl_2_, 0.1 mM EDTA, 2 mM MgCl_2_, 1 mM DTT, 0.5% NP40, cOmplete Protease Inhibitor Cocktail, and Halt^™^ Phosphatase Inhibitor Cocktail) corresponding to five times the cell pellet volume on ice for 5 min. Samples were centrifuged at 3500 × *g* for 15 min at 4°C. The supernatant (cytoplasmic fraction) was then centrifuged at 16 000 × *g* for 10 min and stored at −80°C. The pellet was washed five times with CLB wash buffer (CLB buffer without NP40) and lysed with NLB buffer (nuclear lysis buffer; HEPES, pH 7.9, 20 mM, 10% glycerol, 3 mM EDTA, 150 mM KOAc, 1.5 mM MgCl_2_, 1 mM DTT, 0.1% NP40, cOmplete Protease Inhibitor Cocktail, Halt^™^ Phosphatase Inhibitor Cocktail, and Protector RNase Inhibitor) using pestle homogenization. When no nuclei were visible by microscopic examination, the nuclear fraction was collected by centrifugation at 16 000 × *g* for 10 min at 4°C. Cell fractions were then analyzed by SDS–PAGE and western blotting.

### Cap-association assay using m7GTP-Agarose

To analyze the interaction of EWSR1 with the cap-binding protein complex, HeLa cells were lysed as previously described in [46], using NET2 buffer (Tris–HCl, pH 7.4, 50 mM, 150 mM NaCl, 1 mM phenylmethanesulfonylfluoride, 2 mM benzamidine, and 1% NP-40), and total cell extracts were incubated with m7GTP-Agarose for 2 h at 4°C. Beads were washed three times in NET2 buffer and associated proteins were retrieved in 2× Laemmli buffer and analyzed by SDS–PAGE and western blotting.

### Subcellular fractionation

The S30, S100, R, and RSW fractions were obtained as previously described in [47] with little modifications. Briefly, HEK293 cells, grown to 90% confluence in 10–12 P100 dishes, were washed with ice-cold PBS, and lysed in buffer 1 (Tris–HCl, pH 7.4, 15 mM, 80 mM KCl, 5 mM MgCl_2_, 1% Triton-X-100, and cOmplete Protease Inhibitor Cocktail). Cell debris were removed by two consecutive centrifugations at 14 000 × *g* for 10 min at 4°C. The supernatant of the second centrifugation is the S30 fraction. Centrifugation of the S30 fraction at 95 000 × rpm for 1.5 h using the TLA100.1 rotor led to the S100 fraction (supernatant), and the ribosomes with associated factors (R fraction, pellet). The R-fraction pellet was resuspended in high salt buffer 2 (Tris–HCl, 15 mM, pH 7.4, 500 mM KCl, 5 mM MgCl_2_, 2 mM DTT, and 290 mM sucrose), loaded onto a discontinuous sucrose gradient [bottom layer: 1.5 ml buffer 40% (w/v) sucrose, Tris–HCl, pH 7.4, 15 mM, 500 mM KCl, 5 mM MgCl_2_, and 2 mM DTT] and top layer: 1 ml buffer 20% (w/v) sucrose, Tris–HCl, pH 7.4, 15 mM, 500 mM KCl, 5 mM MgCl_2_, and 2 mM DTT] and centrifuged 95 000 × rpm for 2 h at 4°C using a TLA100.1 rotor. The pure ribosomes pellet (RSW fraction) was resuspended in buffer 1. S30, S100, R, and RSW fractions were analyzed by SDS–PAGE and western blotting.

### GST-fusion proteins purification and pulldowns

Fusion proteins were expressed in One Shot BL21 (DE3) Chemically Competent *Escherichia coli* (Thermo Fischer Scientific, Inc) and purified according to the following protocol. Bacterial culture with appropriate antibiotics was incubated overnight (37°C, 220 × rpm). Next morning, the overnight starter culture was diluted into LB (1:10 dilution) supplemented with appropriate antibiotics and incubated for 2 h (37°C, 220 × rpm). Then, IPTG, dioxane-free (Thermo Fisher Scientific, Inc.) was added to a final concentration of 0.1 mM to induce protein expression and the culture was incubated for 4 h (30°C, 220 × rpm). Cells were harvested by centrifugation at 10 000 × rpm for 10 min at 4°C, then lysed in lysis buffer (1× PBS containing 1% Triton) followed by sonication on ice. The lysate was centrifuged at 10 000 × g for 10 min at 4°C, and the supernatant was incubated with pre-equilibrated Glutathione Sepharose 4B Fast Flow (Cytiva) for 30 min at 4°C with rocking. Beads were collected by centrifugation at 1000 × *g* at 4°C for 3 min, then washed 3× in PBS. After centrifugation, beads can be used immediately for pulldown or stored at −80°C in PBS 10% glycerol.

For pulldown reactions, GST fusion proteins were incubated in IPLS with ribosomal fractions (40S or 60S/80S) collected from polysome profiling. After rocking for 30 min at 4°C, beads were washed 3× in IPLS buffer. Pulldown reactions were then analyzed by SDS–PAGE and western blotting.

### Purification of recombinant proteins for *in vitro* translation assay

To obtain the *pET28a-His-MS2, pET28a-His-MS2-CTD*, or *pET28a-His-MS2 ΔRGG2-ZnF-RGG3* vectors, the *pET28a* plasmid was digested with EcoRI and HindIII, and a BsAI cloning sites was inserted. The resulting plasmid was then digested with BsAI followed by a Golden Gate cloning protocol. Two inserts were introduced: one containing 4 MS2 sites, obtained by annealing two oligonucleotides, and the other generated by PCR, corresponding to the different mutants.

*E. coli* BL21 cells (New England Biolabs, C2527H) containing the resulting vectors were grown to an OD600 of 0.6–0.8 and then induced with 0.5 mM IPTG (Euromedex) for 5 h at 30°C. Cells were lysed using B-Per Bacterial Protein Extraction Reagent buffer (Thermo Scientific, 90084), supplemented with 1 mg/ml Lysozyme (Sigma), 5 U/ml DNAseI (Promega, M6101), and an EDTA-free protease inhibitor cocktail (Merck, 11873580001). The lysate was then centrifuged at 5000 × *g* for 15 min. The supernatant was recovered and incubated with HisPur Cobalt resin (Thermo Scientific, 89965) (previously equilibrated in B-Per Bacterial Protein Extraction Reagent buffer) at 4°C for at least 3 h under gentle shaking. The mixture was then applied to a 2-ml column (Bio-Rad), and the flow-through was collected by gravity. The resin was washed five times with five volumes of native washing buffer (50 mM NaH_2_PO_4_ pH 8.0; 300 mM NaCl; 20 mM imidazole). Bound proteins were eluted with native elution buffer (50 mM NaH_2_PO_4_ pH 8.0; 300 mM NaCl; 500 mM imidazole; protease inhibitor cocktail). Subsequently, the protein was salt-washed with a buffer containing 25 mM Tris–HCl pH 7.5, 50 mM NaCl, 0.1 mM MgCl_2_, 25% glycerol, and protease inhibitor cocktail. Samples were analyzed by 10% SDS–PAGE and stained with Coomassie Blue.

### *In vitro* transcription

RNAs were synthesized by *in vitro* transcription using the EcoRI-linearized *pRenilla* vector, as previously described in [48]. Transcription reactions were performed using 1 μg of linearized DNA template in transcription buffer [40 mM Tris–HCl (pH 7.5), 6 mM MgCl_2_, 2 mM spermidine, and 10 mM NaCl] in the presence of 40 U of T7 RNA polymerase (Promega, P4074), 40 U of RNasin (Promega, N2515), 1.6 mM of each ribonucleotide triphosphate (Promega), and 10 mM DTT. Transcription reactions were carried out at 37°C for 1.5 h, followed by a 30 min treatment with 2.5 U RQ1 DNase (Promega, M6101) to remove the DNA template. Transcribed mRNAs were then isolated using NucleoSpin RNA XS purification columns (Macherey Nagel, 740902.50). The integrity of the *in vitro* transcripts was assessed by electrophoresis on a non-denaturing 1% agarose gel (MetaPhor^®^ Agarose, Lonza Bioscience). RNA concentration was determined by measuring the absorbance with a Nanodrop spectrophotometer. All RNAs were capped with ARCA cap Analog (New England Biolabs, S1411L) and each contains a poly(A) tail of 100 base pairs.

### *In vitro* translation assays

Nuclease-treated Flexi Rabbit Reticulocyte Lysate (RRL, Promega, L4540) was programmed with 10 fmol of *in vitro* synthesized mRNAs in the presence of the indicated amount of recombinant protein. Translation reaction was performed for 45 min at 30°C in a buffer containing 75 mM KCl, 0.5 mM MgOAc, 2 mM DTT, 20 µM of each amino acid, and 5 µl of RRL. Translation reactions were then stopped using Renilla lysis-juice buffer (PJK Biotech, 102531), and Renilla luciferase activity was measured using a Mithras LB940 (Berthold).

### Ribosome profiling (RIBO-seq)

Pellets from HeLa cells transfected with *siCTL* or *siEWSR1* for 48 h, pre-treated with 100 mg/ml cycloheximide, were delivered to OHMX.bio facilities (Ghent, BE). Frozen cell pellets were resuspended with 2 ml of lysis buffer supplemented with 100 mg/ml cycloheximide on ice. Cell lysis was accomplished by passing the suspension through a 18G needle. The cell suspension was incubated in lysis buffer for 10 min and centrifuged twice for complete removal of the cell debris. The clear lysate was aliquoted for total RNA purification with the RNA Clean and Concentrator-5 kit (Zymo Research) and subsequent footprinting. For footprinting and monosome purification, 10 μg of lysate was digested with RNase I (Thermo Fisher Scientific, Inc.) at 21°C for 45 min. The reaction was stopped with addition of Superase-In RNase Inhibitor (Thermo Fisher Scientific, Inc.), and the digested lysate was purified on a MicroSpin S-400 HR Sephacryl column (GE Healthcare). The flow-through was collected and purified with the RNA Clean and Concentrator-5 (Zymo Research). The footprints were visualized on the Agilent Bioanalyzer with the RNA 6000 Nano Kit (Agilent).

Ribosome protected fragments (RPFs) of 28–30 nt were selected by gel electrophoresis on a Novex TBE-Urea gel (15%) (Thermo Fisher Scientific, Inc.). The gel parts corresponding to the fragments of interest were excised and let to diffuse in molecular grade water supplemented with ammonium acetate and SDS overnight at 4°C. The gel slurry was then transferred to CoStar filter tubes and centrifuged. The flow-through was incubated at −80°C for 2 h with glycogen and isopropanol. Following centrifugation at 18 000 × *g* for 20 min, the supernatant was discarded and the pellet was washed with ice-cold 80% EtOH. Pellets were left to dry for 10 min and were resuspended in 15 μl molecular grade water. The quality of the eluted RNA fragments was checked on the Agilent Bioanalyzer using the Small RNA kit (Agilent Technologies).

For library preparation, the RPFs were 3′ dephosphorylated with T4 PNK (New England Biolabs) for 1 h at 37°C to heal the 2′–3′ cyclic phosphate from the RNase I cleavage and were subsequently purified using the Oligo Clean and Concentrator kit (Zymo Research). The 3′ adapter was ligated to the RPFs, and the ligated fragments were purified following the protocol of the Small RNA Library prep kit (Lexogen). After the 5′ phosphorylation of the RPFs and their purification with the Oligo Clean and Concentrator kit, 5′ adapter ligation took place based on the Small RNA Library prep kit. First strand cDNA synthesis was performed according to the Small RNA Library prep kit manual. Ribosomal RNA (rRNA) was depleted with the addition of LNA probes from the QIAseq FastSelect rRNA HMR kit (Qiagen) during the cDNA synthesis reaction. Prior to Indexing PCR amplification, a qPCR was performed to determine the optimal PCR cycle number. During PCR, index primers (Lexogen) were added at both ends of the fragments to produce dual-indexed libraries. The PCR amplification protocol from the Small RNA Library prep kit was followed. Library products were purified with AMPure XP beads, and their quality was determined on the Agilent Bioanalyzer with the DNA High Sensitivity kit (Agilent Technologies). For efficient removal of adapter dimers and excess index primers, the library products were run on a Novex TBE gel (8%) (Thermo Fisher Scientific, Inc.), and the gel part corresponding to the library products was excised and purified. The quality of the final libraries was determined on the Agilent Bioanalyzer with the DNA High Sensitivity kit. The concentration of the libraries was estimated by performing qPCR using a dilution of PhiX Control v3 as standard and samples were pooled in an equimolar way. A test sequencing run (2 × 75 bp) on the Illumina MiSeq platform was performed with the MiSeq Reagent Nano kit v2 (Illumina) to check the quality of the prepared libraries prior to the in-depth sequencing on Novaseq.

For matching mRNA-seq, 100 ng of total RNA material was used. mRNA was first enriched using oligo(dT) beads and was fragmented randomly by adding fragmentation buffer. Then, cDNA was synthesized using random primers, after which a second-strand synthesis buffer, dNTPs, RNase H, and DNA polymerase I were added to initiate the second-strand synthesis. Following end repair and sequencing adaptor ligation, the double-stranded cDNA library was completed through size selection and PCR enrichment. The quality of the final libraries was determined on the Agilent Bioanalyzer with the High Sensitivity DNA kit. The concentration of the libraries was estimated by performing qPCR using a dilution of PhiX Control v3 as standard and samples were pooled in equimolar quantities. Finally, high coverage sequencing was performed on the Novaseq 6000 system with the S4 flowcell and SE50 configuration.

### Bioinformatics analysis

For the mRNA-seq analysis, sequencing adapters were trimmed using SeqPurge v2.0.1 [49]. Ribosomal RNA sequences were filtered out using Bowtie2 v2.3.5 [50]. Reads were then mapped to the GRCh38 genome assembly using STAR v2.5.2 [51] and quantified using featureCounts v2.0.1 [52]. QC for all samples at each step was performed using FastQC v0.11.9 and reports were generated using MultiQC v1.8 [53]. Differential gene expression was performed using the DESeq2 R package [54].

For RIBO-seq experiments, adapters were trimmed and clipped using FASTX v0.0.14. PhiX, rRNA, snRNA, snoRNA, and tRNAs were filtered out, and the remaining reads were mapped to GRCh38 using STAR v2.5.2. Quantification of mapped reads was performed using featureCounts v1.5.2. QC for read quality, and mapping was obtained using FastQC and MultiQC. Mapped RPF were annotated based on Ensembl v100 annotations and their lengths were computated using SAMtools [55]. The 5′ ends of RPFs were used to compute the offset to the P-site position with Plastid using a fixed list of offsets reported in [56]. Offsets were afterwards manually adapted based on subsequent RIBO-QC results, so that the dominant reading frame corresponded to the canonical reading frame for all read lengths. Final offset values were used to pinpoint the reads onto an exact base location, resulting in read counts per genomic base position. BED files were then generated to allow visual inspection of the pinpointed counts in a genome browser.

Differential translation efficiencies (TE) for every mapped gene were calculated using DESeq2 using the ratio between matched RIBO- and RNA-seq read counts as input. Differential expression analysis was performed using DESeq2 with RNA-seq read counts as input. Gene and transcript sequences were extracted using the *biomaRt* R package [57] with Ensembl v100 annotations. Motif counts for EWSR1 and rG4s (G_3-5_-N_1-7_-G_3-5_-N_1-7_-G_3-5_-N_1-7_-G_3-5_) on sequence sets were performed using the *stringr* R package with custom scripts. RBP enrichment analysis was performed using AME (MEME suite) with the RBP database from [58]. Gene ontology enrichment analysis and GSEA were performed in R using the clusterProfiler [59] and fgsea [60] packages, respectively.

Differential pattern analysis was performed using the RiboDiPa R package [61] using aligned BAM files as input with default parameters. Bins of p-site mapping were retrieved from the package output, normalized and visualized using GraphPad Prism 8.

All subsequent data manipulation and analysis were performed in R.

### Lipidomic analysis

An amount of cells containing 10 μg of DNA was homogenized in 700 μl of water with a handheld sonicator and was mixed with 800 μl of HCl (1 M):CH_3_OH 1:8 (v/v), 900 μl CHCl_3_, 200 μg/ml of the antioxidant 2,6-di-tert-butyl-4-methylphenol (BHT; Merck), and 3 μl of SPLASH^®^ LIPIDOMIX^®^ Mass Spec Standard (#330707, Avanti Polar Lipids). After vortexing and centrifugation, the lower organic fraction was collected and evaporated using a Savant Speedvac spd111v (Thermo Fisher Scientific, Inc.) at room temperature and the remaining lipid pellet was stored at −20°C under argon.

Just before mass spectrometry analysis, lipid pellets were reconstituted in 100% ethanol. Lipid species were analyzed by liquid chromatography electrospray ionization tandem mass spectrometry (LC-ESI/MS/MS) on a Nexera X2 UHPLC system (Shimadzu) coupled with hybrid triple quadrupole/linear ion trap mass spectrometer (6500 + QTRAP system; AB SCIEX). Chromatographic separation was performed on a XBridge amide column (150 mm × 4.6 mm, 3.5 μm; Waters) maintained at 35°C using mobile phase A [1 mM ammonium acetate in water- acetonitrile 5:95 (v/v)] and mobile phase B [1 mM ammonium acetate in water-acetonitrile 50:50 (v/v)] in the following gradient: (0–6 min: 0% B to 6% B; 6–10 min: 6% B to 25% B; 10–11 min: 25% B to 98% B; 11–13 min: 98% B to 100% B; 13–19 min: 100% B; 19–24 min: 0% B) at a flow rate of 0.7 ml/min, which was increased to 1.5 ml/min from 13 minutes onwards. SM, CE, CER, DCER, HCER, and LCER were measured in positive ion mode with a precursor scan of 184.1, 369.4, 264.4, 266.4, 264.4, and 264.4, respectively. TAG, DAG, and MAG were measured in positive ion mode with a neutral loss scan for one of the fatty acyl moieties. PC, LPC, PE, LPE, PG, PI, and PS were measured in negative ion mode by fatty acyl fragment ions. Lipid quantification was performed by scheduled multiple reactions monitoring (MRM), the transitions being based on the neutral losses or the typical product ions as described above. The instrument parameters were as follows: Curtain Gas = 35 psi; Collision Gas = 8 a.u. (medium); IonSpray Voltage = 5500 V and −4500 V; Temperature = 550°C; Ion Source Gas 1 = 50 psi; Ion Source Gas 2 = 60 psi; Declustering Potential = 60 V and −80 V; Entrance Potential = 10 V and −10 V; Collision Cell Exit Potential = 15 V and −15 V. The following fatty acyl moieties were taken into account for the lipidomic analysis: 14:0, 14:1, 16:0, 16:1, 16:2, 18:0, 18:1, 18:2, 18:3, 20:0, 20:1, 20:2, 20:3, 20:4, 20:5, 22:0, 22:1, 22:2, 22:4, 22:5, and 22:6 except for TGs which considered: 16:0, 16:1, 18:0, 18:1, 18:2, 18:3, 20:3, 20:4, 20:5, 22:2, 22:3, 22:4, 22:5, and 22:6.

Peak integration was performed with the MultiQuantTM software version 3.0.3. Lipid species signals were corrected for isotopic contributions (calculated with Python Molmass 2019.1.1) and were quantified based on internal standard signals and adheres to the guidelines of the Lipidomics Standards Initiative (LSI) (level 2 type quantification as defined by the LSI). Unpaired *T*-test *P*-values and FDR corrected *P*-values (using the Benjamini/Hochberg procedure) were calculated in Python StatsModels version 0.10.1.

### Transmission electron microscopy (TEM)

Cells were fixed for 60 min at 4°C with 2.5% glutaraldehyde in a Sörensen 0.1 M phosphate buffer (pH 7.4) and post-fixed for 30 min with 2% osmium tetroxide. After dehydration in graded ethanol, samples were embedded in Epon. Ultrathin sections obtained with a Reichert Ultracut S ultramicrotome were contrasted with uranyl acetate and lead citrate. Observations were made with a Jeol JEM-1400 transmission electron microscope at 80 kV and random fields were photographed using an 11-megapixel camera system (Quemesa, Olympus).

### Confocal immunofluorescence

HeLa cells were grown on coverslips and transfected for 48 h with EWSR1#1 or control siRNAs. They were then fixed with 4% (w/v) paraformaldehyde for 20 min at RT and permeabilized with 0.5% Triton X-100 diluted in PBS for 20 min at RT. After a saturation step (1 h incubation with PBS supplemented with 3% BSA and 0.1% tween), cells were incubated with anti-EWSR1 antibody (Santa Cruz Biotechnology, sc39831) for one hour at RT. The coverslips were then rinsed with PBS 0.1% tween and incubated with Alexa secondary antibody (Invitrogen) for 1 h at RT. Cells were then washed with PBS 0.1% tween and stained with Hoechst33342 (Acros Organics) 1/50 000 for 5 min. After rinsing with water, slides were mounted with Mowiol. Samples were analyzed using a Zeiss AiryScan2 confocal microscope (Zeiss).

### Statistics

Unless otherwise indicated, graph values are presented as mean ± SD, calculated for at least three independent experiments (i.e. biological replicates). The significance of overlaps was established with Fischer’s exact *t*-tests using the *GeneOverlap* R package. All other statistical tests were performed with GraphPad Prism 8 and are indicated in each figure legends. *P*-value thresholds are depicted as follows: *P < 0.05; **P < 0.01; ***P < 0.001; ^****^P < 0.0001; ns = not significant.

## Results

### EWSR1 acts as a specific repressor of mRNA translation

To unravel a potential role for EWSR1 in the regulation of translation, we performed parallel transcriptional and ribosome profilings of EWSR1-knocked down cells using paired RNA-seq and RIBO-seq, respectively (Fig. 1A). To this aim, we prepared two independent series of samples from HeLa cells transfected with a control siRNA (*siCTL*) or a validated siRNA against EWSR1 (*siEWSR1#1*) [36]. EWSR1 knockdown (KD) (∼85%) was confirmed by quantitative western blot analysis (Supplementary Fig. S1A and B). Both in the RNA-seq and RIBO-seq datasets, Principal Component Analysis (PCA) revealed separate clustering of the *siEWSR1#1* and *siCTL* samples, indicating that KD of EWSR1 correlates with significant modifications in the cellular transcriptome and translatome (Supplementary Fig. S1C and D). RNA-seq analysis identified a total of 2282 differentially expressed genes (DEGs), which were almost equally distributed between upregulated (*n* = 1189) and downregulated (*n* = 1093) upon EWSR1–KD (FDR < 0.05) (Supplementary Fig. S1E and Supplementary Table S1). Gene Set Enrichment Analysis (GSEA) of KEGG pathways revealed that upregulated DEGs were significantly associated with only two pathways: “*cytokine–cytokine receptor interaction*” (hsa04060) and “*N-glycan biosynthesis*” (hsa00510). Interestingly, downregulated DEGs were associated with multiple cellular processes, including regulation of cytoskeleton [“*regulation of actin cytoskeleton*” (hsa04810) and “*tight junctions*” (hsa04530)], cell metabolism [“*pyruvate metabolism*” (hsa00620), “*glycolysis/gluconeogenesis*” (hsa00010), and “*valine, leucine, and isoleucine degradation*” (hsa00280)], and post-transcriptional gene expression [“*spliceosome*” (hsa03040)] (Supplementary Fig. S1F). In addition, enrichment in pathways such as “*KEGG cell cycle*” (hsa04110) and “*Hallmark G2/M checkpoint*” (M5901) pointed to a pivotal role for EWSR1 in cell cycle progression (Supplementary Fig. S1G). Indeed, EWSR1–KD resulted in reduced cell proliferation (Supplementary Fig. S1H). Compared to control cells, EWSR1–KD cells exhibited an altered cell-cycle distribution, with an increased proportion of cells in G1 phase and reduced proportions in S and G2/M phases, consistent with a delayed in G1/S transition (Supplementary Fig. S1I). The G1 delay upon EWSR1–KD was confirmed by up-regulation of the cyclin-dependent kinase inhibitor p21 (Supplementary Fig. S1J). Altogether, these observations indicate that EWSR1 deficiency impacts proliferation in HeLa cells.

**Figure 1.**
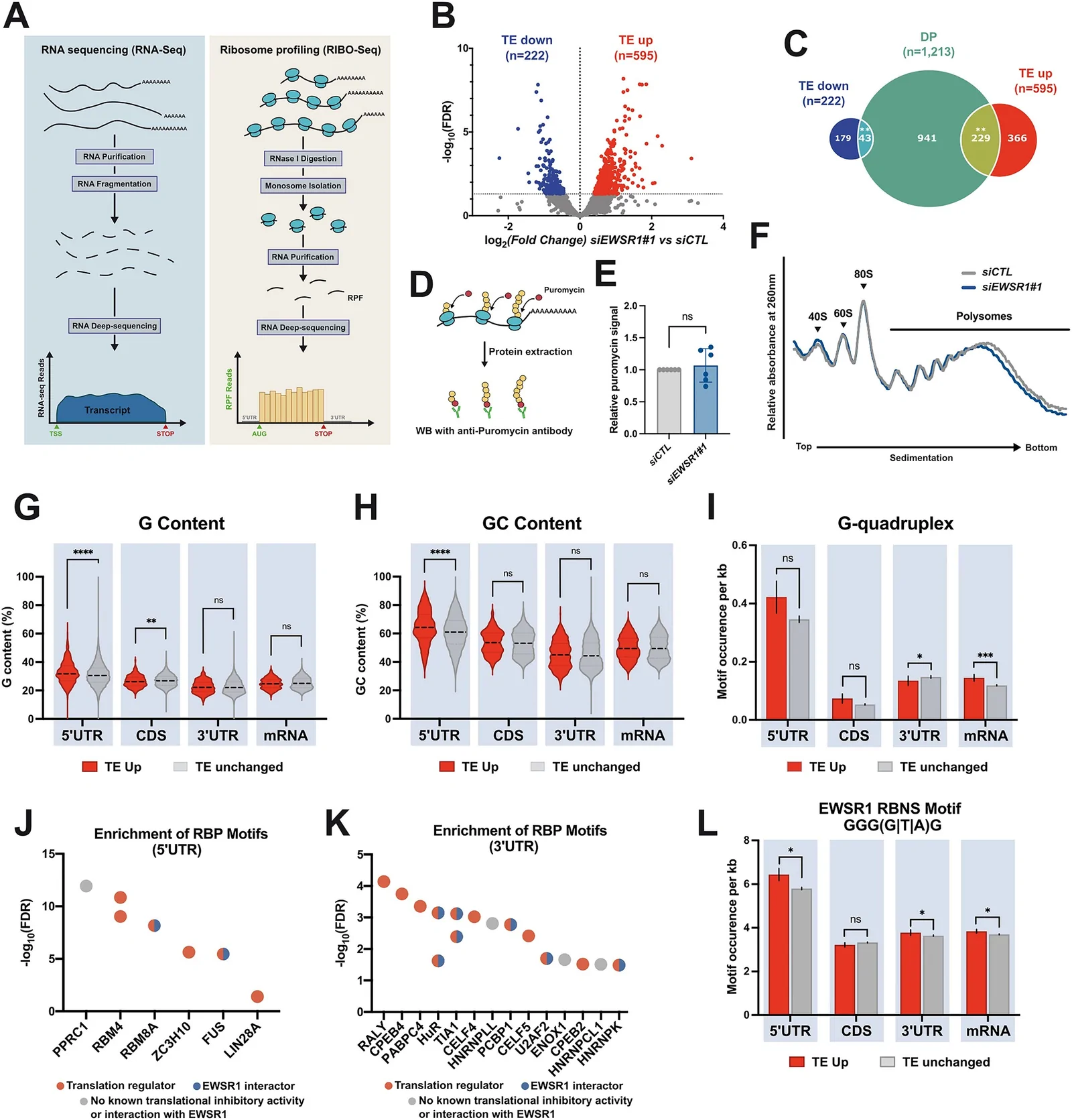
EWSR1 represses translation of a specific set of mRNAs. (**A**) Overview of the RIBO-seq analysis workflow. RPF: ribosome protected fragments, TSS: transcription start site, TES: transcription end site. (**B**) Volcano plot of mRNAs with significantly upregulated (red dots, TE up) and downregulated (blue dots, TE down) translation efficiency (TE) upon EWSR1–KD in HeLa cells (*n* = 2 independent experiments, FDR = 0.05). The numbers of TE up or down mRNAs are indicated. Gray dots correspond to mRNAs that did not show statistically significant differences in TE. (**C**) Venn diagram showing overlap between mRNAs with significantly differential pattern (DP), as identified by RiboDiPA (*n* = 1213) and TE up or TE down mRNAs, as identified by RIBO-Seq (*n* = 595 and *n* = 222, respectively) in Hela cells treated for 48 h with *siEWSR1 versus siCTL*; ***P < 0.001. (**D**) Schematic representation of the SUrface SEnsing of Translation (SUnSET) assay principle. (**E**) Quantification of puromycin incorporation as revealed by western blotting and signal densitometry analysis using ImageJ after normalization to a loading control (β-tubulin). Samples are total cell lysates from HeLa cells treated with *siCTL* or *siEWSR1#1* for 48 h. Results are shown as means ± SD (*n* = 6 independent experiments). ns = not significant compared to the *siCTL* condition by one sample *t* test. (**F**) Polysome profilings of HeLa cells on a 15%–50% sucrose gradient show no differences between 48 h *siCTL* (grey profile) or *siEWSR1#1* (blue profile) conditions. The positions of 40S, 60S, monosomes (80S), and polysomes are indicated. Curves are average from three independent polysome gradients. (**G–I**) Features of EWSR1 TE up targets (Supplementary Table S2). Comparison of G content (G), GC content (H), and G-quadruplex (I) between unchanged (*n* = 15 694) and TE up mRNAs (*n* = 595) in the 3′ and 5′UTRs, CDS regions, and across the entire sequence. *P < 0.05, **P < 0.01, ***P < 0.001, ^****^P < 0.0001, ns = not significant using Wilcoxon–Mann–Whitney test. Results are shown as mean ± SEM (*n* = 2 independent experiments). (**J, K**) RBP motif enrichment analysis in 5′ (J) and 3′UTRs (K) of EWSR1 TE up targets. UTR sequences were screened for RBP motifs using AME from the MEME suite. Enriched motifs with an adjusted *P*-value (padj) < 0.05 are shown. Motifs of RBPs known as translational regulators or/and as EWSR1 interacting partners are indicated in red or/and blue dots, respectively. Gray dots indicate motifs associated with RBPs not known to regulate translation or associate with EWSR1. (**L**) Comparison of EWSR1 RBNS motif (occurrences per kb) in the 5′UTRs, CDS, 3′UTR and full-length sequences of unchanged and TE up mRNAs. *P < 0.05, ns = not significant using Wilcoxon–Mann–Whitney test. Results are shown as ± SEM (*n* = 2 independent experiments).

In parallel to RNA-seq, we also assessed changes in the translational landscape of EWSR1–KD cells, using RIBO-Seq (Fig. 1A). Length distribution of ribosome-protected fragments (RPFs) in RIBO-seq dataset was centered on 29–30 nucleotides, which corresponds to the fragment size protected by translating ribosomes. In addition, RPFs showed predominant phase 0 enrichment, as expected from high quality ribosome profiling experiments (Supplementary Fig. S1K). The majority of RIBO-seq reads mapped to protein-coding mRNA (56.96%), while the remaining mapped to other RNA biotypes including lncRNAs (20.85%) and sncRNAs, such as miRNAs (0.88%), snRNAs (0.19%), and snoRNAs (0.12%) (Supplementary Fig. S1L). Although the high proportion of reads mapping to lncRNAs might look surprising, it has been reported before that lncRNAs can be bound by ribosomes [56]. When focusing on mRNAs, we found that RPF reads almost exclusively mapped to the CDS region (97.11%), while the reads mapping to untranslated regions or introns each represented <1% of RPFs (0.31% to 3′UTRs, 0.58% to 5′UTRs, and 0.95% to introns) (Supplementary Fig. S1M). Altogether, these analyses support the robustness and quality of our RIBO-seq dataset.

To assess changes in translation efficiency (TE) following EWSR1–KD, we combined RIBO-seq and RNA-seq data. TE corresponds to the density of RPF normalized to mRNA abundance and provides a relative measure of translational engagement rather than a direct measure of absolute protein production [62]. Genome-wide analysis of TE revealed significant changes in translation of individual mRNA between control and EWSR1–KD cells. From a total of 16 511 mRNAs for which the coverage was sufficient to compute a TE value, we identified a list of 595 mRNAs showing a significant increase in TE upon EWSR1–KD (log_2_FoldChange > 0 and adjusted *P*-val < 0.05, hereafter “TE up genes”) (Fig. 1B and Supplementary Table S2). In contrast, only 222 genes had a decreased TE upon EWSR1–KD (log_2_FoldChange < 0 and adjusted *P*-val < 0.05, hereafter “TE down genes”). This raised the exciting possibility that EWSR1 could carry roles in translational regulation. Since we found that almost three times (i.e. 595/222; 2.68-fold) more mRNAs showed increased TE following EWSR1–KD, we hypothesized that EWSR1 preferentially plays a repressive role in mRNA translation. To complement our TE analysis, we next used RiboDiPA, a method using differences in ribosome binding patterns along transcripts to detect translational changes between conditions [61]. Using this method, we identified 1213 mRNAs with significantly differential pattern (DP) of RPF distribution between *siCTL* and *siEWSR1* conditions (Supplementary Table S3). Interestingly, 38% (229/595) of the TE up mRNAs were also found among the transcripts with differential RPF pattern (DP) (Fig. 1C and Supplementary Fig. S1N). In contrast, only 19% (43/222) of TE down were also identified by DP analysis. In conclusion, both differential TE and DP analyses demonstrate that EWSR1–KD correlates with extensive changes in the cellular translational landscape.

To further investigate the effects of EWSR1–KD on global translation, we used a Surface Sensing of Translation (Sunset) assay to monitor and quantify global changes in protein synthesis by detecting puromycin-labeled polypeptides [63] (Fig. 1D). Quantification of puromycin incorporation by western blotting did not show any significant changes in protein synthesis after EWSR1–KD, suggesting that EWSR1 is not a general regulator of translation (Fig. 1E and Supplementary Fig. S1O). To confirm this, we performed polysome profile analysis by sucrose density gradient fractionation. We observed no significant alteration of the polysome profiles in *siEWSR1*-transfected HeLa cells, again indicating that EWSR1–KD has no effect on global translation (Fig. 1F). Taking together, our results thus suggest that EWSR1 regulates translation of a specific subset of mRNAs.

### EWSR1 translational targets exhibit specific sequence features

The large imbalance in favor of TE up versus TE down transcripts highlighted by our differential TE analysis (Fig. 1B) suggests that EWSR1 might preferentially act as a repressor of translation. To gain molecular insights on how EWSR1 represses translation of specific transcripts, we investigated the sequence features of the 595 TE up mRNAs (Supplementary Table S2). Compared to a control set of transcripts whose TE is unaffected by EWSR1–KD (hereafter “TE unchanged,” *n* = 15 694), we observed that 5′UTRs of the TE up mRNAs had a significantly higher G- and GC-contents (Fig. 1G and H). This was not the case for TE down mRNAs (Supplementary Fig. S1P and Q). Furthermore, EWSR1 TE up mRNAs also showed higher occurrence of G-quadruplexes (Fig. 1I). In contrast, TE down mRNAs were significantly depleted of G-quadruplexes (Supplementary Fig. S1R). Enrichment for rG4 in TE up mRNA was also confirmed independently using the rG4 and rG4-binding proteins database QUADRatlas, which identified 348 (58.5%; 348/595) experimentally validated rG4-containing mRNA and 569 (95.6%; 569/595) predicted rG4-containing mRNA among the TE up transcripts [64]. Considering previous reports showing that the RGG domains of EWSR1 specifically bind to rG4s, these observations were especially interesting and suggested that EWSR1 might repress translation through association with its target mRNAs [65]. Numerous studies have highlighted the key roles played by 5′ and 3′UTRs in the control of mRNA translation [66, 67]. To further investigate the model of EWSR1-mediated translational repression, we performed an RNA-binding motif enrichment analysis in 5′ and 3′UTRs of TE up mRNAs using AME from the MEME-suite [68] based on the sequence specificities of 205 RBPs defined by unbiased, *in vitro* competitive binding analysis [69]. We found that 5′UTRs of TE up transcripts were enriched in motifs associated with PPRC1, RBM4, RBM8A, ZC3H10, LIN28A, and the EWSR1 paralog FUS (Fig. 1J). The 3′UTRs of TE up mRNAs were enriched in a larger number of RBP motifs, including those of CPEB4, HuR, TIA1, and PCBP1 (Fig. 1K). Remarkably, the majority of these RBPs have been previously identified as regulators of translation and/or EWSR1 interactors [31, 70–81, 82]. This suggests that EWSR1 might repress the translation of its target mRNAs by collaborating with known translation inhibitors, for instance by enhancing their recruitment onto EWSR1 translational targets. Alternatively, some EWSR1-partner RBPs may increase EWSR1 binding to its target mRNAs. Therefore, we specifically searched for potential EWSR1-binding motifs in TE up mRNAs. Although EWSR1 is thought to bind RNA through multiple modes, a recent study combining *in vitro* RNA Bind-N-Seq (RBNS) analysis with enhanced CLIP (eCLIP) identified GGG(G|T|A)G as being the motif most strongly associated with EWSR1 binding [83]. We found this motif to be significantly enriched in TE up mRNAs, specifically in the 5′ and 3′UTRs (Fig. 1L). In contrast, the 3′UTR of TE down mRNAs were significantly depleted for the EWSR1 RBNS motif (Supplementary Fig. S1S). These analyses point towards a model in which mRNAs whose translation is inhibited by EWSR1 harbor specific structural and sequence features such as high GC- and G-contents, high incidence of rG4 structures and GGG(G|T|A)G motifs, all of which are compatible with the association of EWSR1 with its translational targets.

### EWSR1 binds its translational targets and represses translation via its RGG2–ZnF–RGG3 region

To support the model whereby EWSR1 binds to specific mRNAs to repress their translation, we sought to test its ability to associate with TE up transcripts. To this aim, we identified EWSR1 RNA binding targets based on previously published CLIP-seq data [20, 83, 84]. We compiled a “high-confidence dataset” of 2 778 EWSR1-bound mRNAs that were identified in the three CLIP-seq datasets (Supplementary Table S4), of which 2717 were detected in our RIBO-seq analysis. Almost one fourth (22.2%; 132/595) of the TE up mRNAs were found in the set of high-confidence EWSR1-bound mRNAs, an overlap that was highly significant (Fig. 2A). Because these mRNAs are bound by EWSR1 and their translation is increased in the absence of EWSR1, we defined these 132 overlapping transcripts as putative “EWSR1 translational targets.” To cross-validate the binding of EWSR1 to these mRNAs in HeLa cells, we performed RNA immunoprecipitation (RIP)-qPCR experiments on six randomly selected “EWSR1 translational targets” (i.e. *SEC61G, ADARB1, KDELR1, CHD2, ZNF664*, and *RPL32)*. By comparison to *GAPDH*, we found that 5 out of the 6 tested mRNAs (i.e. *SEC61G, ADARB1, KDELR1, CHD2*, and *ZNF664)* were significantly enriched in the EWSR1 IP (Fig. 2B). This indicates that EWSR1 can associate with mRNAs whose translation is increased following EWSR1 depletion.

**Figure 2.**
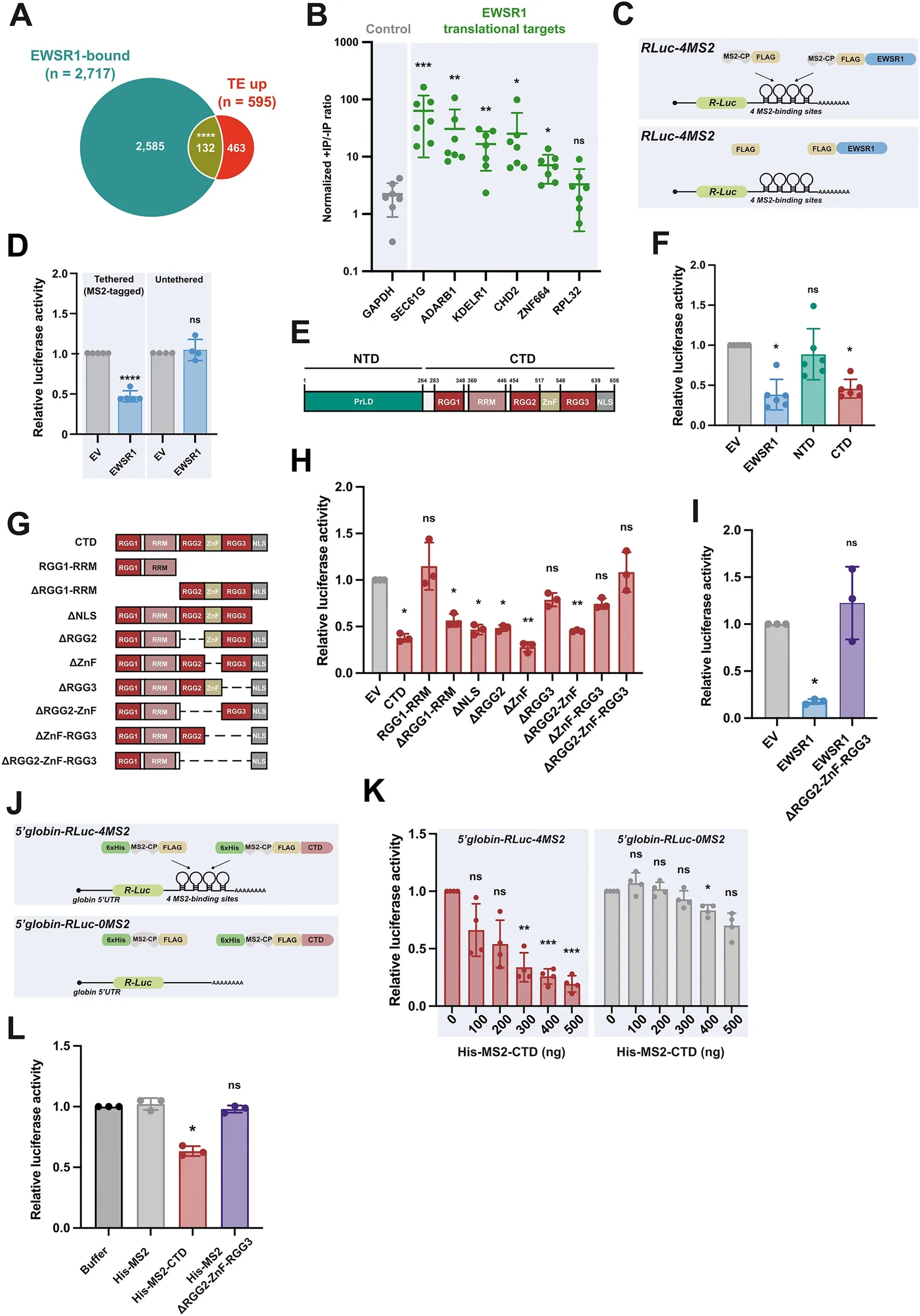
EWSR1 binds to its translational targets and represses translation via the RGG2–ZnF–RGG3 region within its CTD. (**A**) Venn diagram showing a significant overlap between a high confidence dataset of EWSR1-bound transcripts curated from literature (*n* = 2717) and TE up genes (*n* = 595). ^****^P < 0.0001 using Fisher’s exact test. (**B**) RIP-qPCR enrichment of the indicated TE up mRNA targets following EWSR1 immunoprecipitation. Enrichment values were calculated as the ratio of EWSR1 versus control immunoprecipitation (IP+/IP−) normalized to 18S rRNA. Data are presented as means ± SD from seven independent experiments. Statistical analysis was performed on log_2_-transformed normalized enrichment values using repeated-measures one-way ANOVA followed by Dunnett’s multiple-comparisons test against *GAPDH*. *P < 0.05, **P < 0.01, ***P < 0.001, ns = not significant. (**C**) Representation of the *R-Luc-4MS2* tethering assay. Fusing FLAG–EWSR1 to the MS2-CP allows its specific tethering to MS2-binding sites located in the 3′UTR of the Renilla luciferase (RLuc) mRNA reporter. A CMV bidirectional promoter controls the transcription of both Firefly and Renilla Luciferase genes. (**D**) Luciferase tethering assays in HeLa cells transfected with constructs coding for MS2-CP-tagged (tethered) or untagged (untethered) versions of FLAG-EV (Empty vector) or FLAG–EWSR1 together with *RLuc-4MS2* or *RLuc-0MS2* reporters. Results are means of normalized RLuc/FLuc activities [(RLuc-4/FLuc)/(RLuc-0/FLuc)] relative to the FLAG-EV condition ± SD (n ≥ 4 independent experiments). ^****^P < 0.0001, ns = not significant compared to the FLAG-EV condition by one sample *t-*test. (**E**) Domain organization of EWSR1. NTD: N-terminal domain, CTD: C-terminal domain, PrLD: Prion-like domain, RRM: RNA-recognition motif, RGG: arginine–glycine–glycine-rich motif, ZnF: Zinc-finger, NLS: nuclear localization signal. Positions of amino acids are indicated. (**F**) Luciferase tethering assays in HeLa cells transfected with constructs encoding MS2-CP-fused versions of FLAG-EV (Empty vector) or FLAG-tagged EWSR1, NTD or CTD together with *RLuc-4MS2* or *RLuc-0MS2* reporters. Results are means of normalized RLuc/FLuc activities [(RLuc-4/FLuc)/(RLuc-0/FLuc)] relative to the FLAG-EV condition ± SD (*n* = 6 independent experiments). Statistical analyses were performed on the raw RLuc/FLuc ratios using repeated-measures one-way ANOVA followed by Dunnett’s multiple-comparisons test against EV. *P < 0.05, ns = not significant. (**G**) Schematic of CTD-deletion mutants. (**H**) Luciferase tethering assays in HeLa cells transfected with constructs encoding MS2-CP-fused versions of FLAG-EV (Empty vector) or FLAG-tagged full-length or deletion mutants of EWSR1 CTD, together with *RLuc-4MS2* or *RLuc-0MS2* reporters. Results are means of normalized RLuc/FLuc activities [(RLuc-4/FLuc)/(RLuc-0/FLuc)] relative to the FLAG-EV condition ± SD (*n* = 3 independent experiments). Statistical analyses were performed on the raw RLuc/FLuc ratios using repeated-measures one-way ANOVA followed by Dunnett’s multiple-comparisons test against EV. *P < 0.05, **P < 0.01, ns = not significant. (**I**) Luciferase tethering assays in HeLa cells transfected with constructs encoding MS2-CP-fused versions of FLAG-EV (Empty vector) or FLAG-tagged full-length EWSR1 or the indicated EWSR1 deletion mutant, together with *RLuc-4MS2* or *RLuc-0MS2* reporters. Results are means of normalized RLuc/FLuc activities [(RLuc-4/FLuc)/(RLuc-0/FLuc)] relative to the FLAG-EV condition ± SD (*n* = 3 independent experiments). Statistical analyses were performed on the raw RLuc/FLuc ratios using repeated-measures one-way ANOVA followed by Dunnett’s multiple-comparisons test against EV. *P < 0.05, ns = not significant. (**J**) Representation of the *5′-globin-RLuc-4MS2* and *5′-globin-RLuc-0MS2* reporter mRNAs. (**K**) *In vitro* luciferase tethering assay in RRL programmed with the *5′-globin-RLuc-4MS2* or *5′-globin-RLuc-0MS2* reporter mRNAs in the presence of the indicated amounts of recombinant His-tagged EWSR1 CTD fused to MS2-CP. Reporter activities were normalized to the corresponding 0 ng condition within each experiment. Results are means ± SD from four independent experiments. Statistical analysis was performed using two-way repeated-measures ANOVA with reporter and dose as factors, followed by Šídák’s multiple-comparisons test comparing each dose to the corresponding 0 ng control for each reporter. *P < 0.05, **P < 0.01, ***P < 0.001, ns = not significant. (**L**) *In vitro* luciferase tethering assay in RRL programmed with the *5′-globin-RLuc-4MS2* reporter mRNA in the absence (Buffer) or presence of 250 ng of the His tag fused to MS2 (His-MS2) or similar amounts of recombinant His-tagged wild-type or mutant EWSR1 CTD. Results are means of normalized RLuc activity relative to the buffer condition within each experiment ± SD from three independent experiments. Statistical significance was assessed by one-way ANOVA followed by Dunnett’s multiple-comparisons test with His-MS2 as the control condition. *P < 0.05, ns = not significant.

To validate this model, we used a dual-luciferase translation reporter assay [85]. This mRNA tethering assay is based on a *Renilla luciferase* (*RLuc*) reporter mRNA harboring four repeats of the binding sequence for the bacteriophage MS2–coat protein (MS2-CP) in its 3′UTR and designated *RLuc-4MS2* (Fig. 2C). Renilla luciferase activity was normalized to an internal Firefly luciferase (FLuc) control to account for variations in transfection efficiency and reporter expression. Tagging EWSR1 with the MS2-CP peptide allows its specific tethering to the 3′UTR of the *RLuc-4MS2* reporter transcript. Results are expressed relatively to an untethered *RLuc* mRNA lacking MS2-binding sites (*RLuc-0MS2*), which is used as a control for tethering (Supplementary Fig. S2A). To test the effect of EWSR1 on the translation of the *RLuc* reporter mRNA, we transfected HeLa cells with FLAG-tagged constructs encoding either MS2-CP alone (MS2-empty vector or MS2-EV), as control or MS2-CP fused to the full-length EWSR1 construct (MS2–EWSR1), together with the *RLuc-0MS2* (as the control reporter) or *RLuc-4MS2* (as the tethered reporter). Compared to MS2-CP alone, tethering of MS2–EWSR1 to the *RLuc-4MS2* transcript specifically reduced expression of the RLuc protein by more than half (i.e. 2.17-fold or 54%) (Fig. 2D). Importantly, a non-MS2-fused FLAG–EWSR1 had no effect in the same assay, indicating that the observed reduction in RLuc protein levels by EWSR1 requires its tethering to the reporter mRNA. Tethering of EWSR1 did not reduce the steady-state levels of the *RLuc-4MS2* transcript, suggesting that EWSR1 inhibits the translation of the *Renilla* luciferase, without affecting the abundance of the encoding transcript (Supplementary Fig. S2B). Altogether, these observations support a model whereby EWSR1 represses translation through association with its target mRNAs.

To gain further insights into its translational function, we sought to pinpoint which region of EWSR1 was implicated in translational repression. To this aim, we first tested independently its prion-like N-terminal region (NTD, N-terminal domain, aa 1–264) and C-terminal RNA-binding domain (CTD, C-terminal domain, aa 266–656) in the MS2-tethering reporter assay (Fig. 2E). Similarly to full-length EWSR1, we found that tethering of the CTD led to a 2-fold reduction (i.e. 2.18-fold or 54.1%) in the expression of the *RLuc* reporter, while having no effect on the abundance of the *RLuc* transcript (Fig. 2F and Supplementary Fig. S2C). In contrast, the NTD had no significant effect on RLuc expression, although EWSR1 domains were expressed at comparable levels in the assay (Supplementary Fig. S2D). As for EWSR1, the translational inhibition of the reporter by EWSR1 CTD was not observed when transfecting an untethered version of the protein (i.e. not fused to MS2-CP), further demonstrating that EWSR1-mediated translational repression requires its tethering to the target transcript (Supplementary Fig. S2E). To further support these observations, we used a different *RLuc* reporter, bearing six MS2-binding sites in its 3′UTR [86]. Using this alternative reporter, the EWSR1 CTD exhibited an even stronger effect, reducing RLuc expression by >80% (i.e. 5.9-fold or 83%) (Supplementary Fig. S2F). Altogether, these results identify the CTD as the region of EWSR1 responsible for its translational repressive function.

To narrow down the region of EWSR1 implicated in the inhibition of translation, we generated a series of nine mutants of the CTD region, each lacking specific structural and/or functional domains and tested them in the *RLuc-4MS2* tethering reporter assay (Fig. 2G). Among the tested CTD mutants, which were all expressed at comparable levels (Supplementary Fig. S2G), two had completely lost the ability to repress the synthesis of RLuc, namely the RGG1–RRM and ΔRGG2-ZnF-RGG3 mutants (Fig. 2H). These translationally inactive mutants both lacked a region of the CTD spanning from the second to the third RGG motifs and including the ZnF (i.e. the RGG2–ZnF–RGG3 region). The absence of the ZnF alone had no effect, as the ΔZnF mutant retained full repressive activity. The presence of either RGG2 or RGG3 (as in the ΔRGG2–ZnF or ΔZnF–RGG3 mutants) resulted in a modest and reproducible trend towards translational repression, although this effect did not reach statistical significance. In agreement with these observations, an EWSR1 mutant protein lacking the RGG2–ZnF–RGG3 had completely lost the ability to repress RLuc expression (Fig. 2I and Supplementary Fig. S2H). To further investigate the translational repressive activity of EWSR1, we tested the ability of its CTD region to interfere with translation of an exogenous reporter transcript *in vitro* using the untreated RRL system [87]. For this, we *in vitro* transcribed a *RLuc* mRNA harboring a *globin* 5′-UTR and a 3′UTR containing either 4 or no MS2 binding sites (*5′globin-RLuc-4MS2* and *5′globin-RLuc-0MS2*, respectively) (Fig. 2J). Then, we assessed the effect of adding recombinant EWSR1 CTD fused to 6xHis and MS2-CP (His–MS2–CTD) in RRL programmed with the *5′globin-RLuc* mRNA. We used the CTD because it inhibited translation in cells, similarly to full-length EWSR1 (Fig. 2F) and was easier to express in bacteria and purify. The results show that translation of *RLuc* was inhibited by addition of increasing amounts of recombinant His–MS2–CTD in a dose response manner (Fig. 2K). Addition of as little as 100 and 200 ng of His–MS2–CTD lead to approximately 34% (33.9%) and 46% (45.9%) inhibition of translation, respectively. As a control, translation from the *5′globin-RLuc-0MS2* transcript remained unaffected in these conditions, being only slightly reduced with the highest amounts of His–MS2–CTD (400 and 500 ng). Next, we sought to use this assay to validate the importance of the RGG2–ZnF–RGG3 region of EWSR1 in its ability to repress translation. As expected, adding 250 ng of purified His–MS2–CTD significantly inhibited translation of the *5′globin-RLuc-4MS2* reporter transcript (Fig. 2L). Addition of similar quantities of the ΔRGG2-ZnF-RGG3 CTD mutant fused to His–MS2-CP had no effect, thus confirming that the translation inhibition activity of EWSR1 lies within its RGG2–ZnF–RGG3 region (Fig. 2L and Supplementary Fig. S2I). Altogether, these results support a model in which EWSR1 represses translation upon association with a target transcript and involves its RGG2–ZnF–RGG3 region.

Next, we sought to test the possibility that EWSR1 might inhibit translation by interfering with initiation, the rate-limiting step of translation. First, we tested whether EWSR1 might affect cap recognition using a cap-binding assay. While we were able to efficiently pulldown several translation initiation factors, we found no evidence that endogenous EWSR1 associates with m^7^GTP-Agarose (Supplementary Fig. S3A). Of note, knockdown of EWSR1 had no effect on binding of various eIF4F components to the immobilized m^7^GTP. Next, we tested the effect of EWSR1 on cap-independent translation driven by internal ribosome entry sites (IRES). Different classes of viral IRES elements exist, categorized based on their requirements for eukaryotic initiation factors (eIFs). For example, the poliovirus (PV) IRES requires most of the canonical eIFs, with the notable exception of the cap-binding protein eIF4E. In contrast, the hepatitis C virus (HCV) IRES functions independently of eIF4E, eIF4G, and eIF4A. Remarkably, the IRES from the Cricket Paralysis Virus (CrPV) can initiate translation without the need for any eIFs. We took advantage of this diversity to determine whether EWSR1 targets a specific eIF or a particular step of the initiation process. We cloned the sequences corresponding to the PV or CrPV IRES in the 5′UTR of a *RLuc* reporter with 8 MS2 loops in its 3′UTR (i.e*. PV-RLuc-8MS2* and *CrPV-RLuc-8MS2*, respectively). By comparing with an untethered control *RLuc* mRNA lacking MS2-binding sites (i.e. PV-*RLuc-0MS2* and *CrPV-RLuc-0MS2*), we tested the effect of tethering EWSR1 or its CTD on IRES-driven *RLuc* translation in HeLa cells (Fig. 3A). Tethering of full length EWSR1 or its CTD significantly reduced translation from both the *CrPV-RLuc-8MS2* and *PV-RLuc-8MS2* reporters, similarly to our observations with cap-dependent reporters (Fig. 3B and Supplementary Fig. S3B,C). Importantly, the ability of EWSR1 to repress IRES-dependent translation was confirmed *in vitro* using the RRL reporter system, in which EWSR1 CTD but not its ΔRGG2-ZnF-RGG3 mutant repressed translation when tethered to the 3′UTR of *RLuc* reporters driven by the CrPV or the HCV IRES. These findings indicate that EWSR1-mediated translational repression does not require canonical ribosomal scanning (Fig. 3C). To confirm this, we used a previously described *RLuc-6xMS2* reporter whose translation occurs via a scanning‐independent mechanism, using a translation initiator of short 5′UTR (TISU) element (*TISU-RLuc-6xMS2*) [86] (Fig. 3D). We found that EWSR1 CTD inhibited expression of RLuc from this reporter (Fig. 3E). Altogether, these observations suggest that inhibition of translation by EWSR1 is independent of cap recognition or 5′UTR scanning by the 43S pre-initiation complex (PIC).

**Figure 3.**
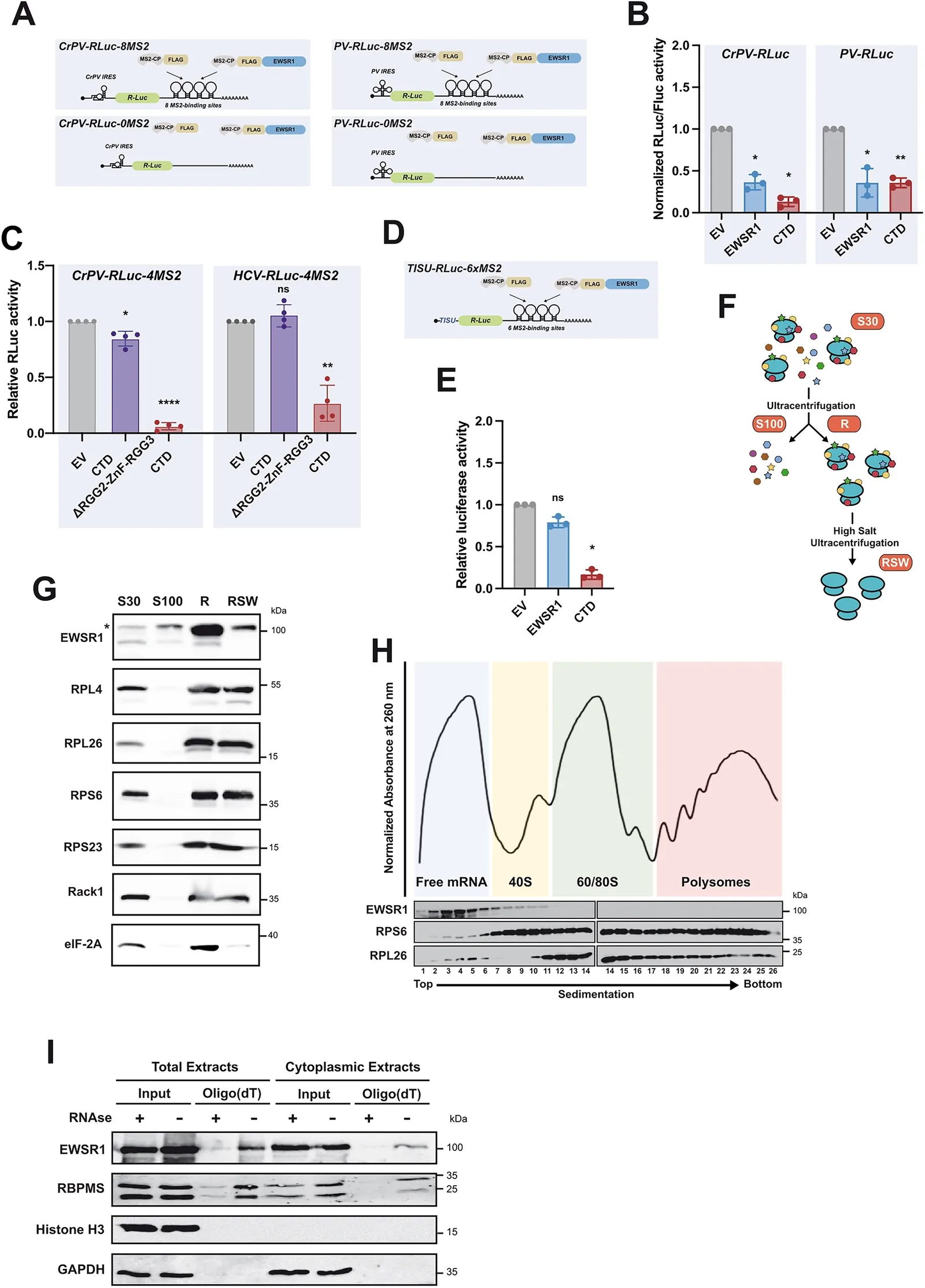
EWSR1 represses translation independently of canonical cap recognition and 43S scanning. (**A**) Representation of the IRES-dependent translation assays using the *CrPV-RLuc-8MS2* and *CV-RLuc-8MS2* reporters. Fusing FLAG–EWSR1 to the MS2-CP allows its specific tethering to MS2-binding sites located in the 3′UTR of the *Renilla luciferase* mRNA reporter. Corresponding reporters lacking MS2-binding sites in their 3′UTR and MS2-CP–FLAG constructs are used as controls (*CrPV-RLuc-0MS2* and *CV-RLuc-0MS2*, respectively). A CMV bidirectional promoter controls the transcription of both Firefly and Renilla Luciferase genes. (**B**) Luciferase tethering assays in HeLa cells transfected with constructs coding for MS2-CP-tagged version of FLAG-EV (Empty vector) or FLAG-tagged EWSR1 or CTD together with *PV-RLuc* or *CrPV-RLuc* reporters illustrated in (A). Results are means of normalized RLuc/FLuc activities [(RLuc-8/FLuc)/(RLuc-0/FLuc)] relative to the FLAG-EV condition ± SD from three independent experiments. Statistical analyses were performed on the raw RLuc/FLuc ratios using repeated-measures one-way ANOVA followed by Dunnett’s multiple-comparisons test against EV; *P < 0.05, **P < 0.01. (**C**) *In vitro* luciferase tethering assay in RRL programmed with a *CrPV-RLuc-4MS2* or a *HCV-RLuc-4MS2* reporter mRNA in the presence of 0 ng (buffer) or 250 ng of recombinant His-tagged wild-type or ΔRGG2-ZnF-RGG3 mutant EWSR1 CTD, fused to MS2. Results are means of normalized RLuc activity relative to the buffer condition ± SD from four independent experiments. Statistical analyses were performed on the raw Luciferase activities using repeated-measures one-way ANOVA followed by Dunnett’s multiple-comparisons test against the buffer conditions; *P < 0.05, **P < 0.01, ns = not significant. (**D**) Representation of the 43S scanning-independent *TISU-RLuc-6xMS2* reporter mRNA. (**E**) Luciferase tethering assays in HeLa cells transfected with constructs coding for MS2-CP-tagged version of FLAG-EV (Empty vector) or FLAG-tagged full-length EWSR1 or its CTD together with the *TISU-RLuc-6xMS2V* reporter illustrated in (D). Results are means of normalized RLuc/FLuc activities relative to the FLAG-EV condition ± SD from three independent experiments. Statistical analyses were performed on the raw RLuc/FLuc ratios using repeated-measures one-way ANOVA followed by Dunnett’s multiple-comparisons test against the FLAG-EV condition; *P < 0.05, ns = not significant. (**F**) Subcellular fractionation protocol. S30: total cell lysate, S100: ribosome-depleted cell lysates, R: ribosomes with associated factors, RSW: high-salt washed ribosomes without associated factors. (**G**) Western blotting of S30, S100, R, and RSW fractions, as described in (F). The band corresponding to EWSR1 is indicated by an asterisk. (**H**) Absorbance profiles at 260 nm (top) and western blot analysis (bottom) of sucrose density fractionation of HeLa cytoplasmic extracts. The “free mRNA” (i.e. mRNA not associated with ribosomes) pool corresponds to fractions 1 to 6 (light blue), free 40S particles are found in fractions 7 to 11 (yellow), free 60S and 80S monosomes co-sediment in fractions 12 to 16 (light green), and polysomes are found in fractions 17 to 26 (pink). The distributions of EWSR1, RPS6 (40S component), and RPL26 (60S component) throughout the different fractions were assessed by western blotting. (**I**) Western blot analysis of EWSR1 in oligo(dT)-RNA-bound proteins isolated from total or cytoplasmic extracts of HeLa cells transfected with FLAG–EWSR1. Cells were UV cross-linked and treated (+) or not treated (−) with RNase A before pulldown. RBPMS, a well-characterized RBP, serves as a positive control, while GAPDH and Histone H3 are used as negative controls.

### EWSR1 is found in non-translating mRNPs

The above findings led us to investigate the possibility that EWSR1 might regulate translation by engaging in interactions with ribosomes. In agreement with this hypothesis, we verified the presence of EWSR1 in the cytoplasm of HeLa cells using biochemical fractionation (Supplementary Fig. S3D). Confocal microscopy analysis revealed a discrete and punctuated pattern for cytoplasmic EWSR1 (Supplementary Fig. S3E). Next, we conducted a multistep fractionation protocol to obtain the S100 (fraction free of ribosomes), R (ribosomes with associated factors), and RSW (Ribosome Salt Wash, i.e. ribosomes with no associated factors) fractions from total cell extracts (S30) (Fig. 3F) [88]. As expected, core ribosomal proteins such as 40S (RPS6, RPS23, and RACK1) and 60S (RPL26 and RPL4) components were specifically detected in the R and RSW fractions, but not in the S100 fraction (Fig. 3G). The initiation factor eIF-2A was enriched in the R fraction but barely visible in the RSW fraction, showing that associated factors such as initiation factors were efficiently detached from the ribosomes by the high salt wash between the R and RSW steps, as expected. A large portion of EWSR1 was detected in the R fraction, demonstrating that it mostly associates with ribosomes. Because only a small proportion of EWSR1 was found in the RSW fraction, we concluded that although EWSR1 sediments with the ribosomes, it is not an integral component of the ribosomal particles.

These results prompted us to determine the type of ribosomal particle interacting with EWSR1. To this aim, we first resolved HeLa cells cytoplasmic extracts by ultracentrifugation through a linear sucrose density gradient. This approach generated a profile including fractions corresponding to mRNA outside of ribosomes (also known as the “free” mRNA pool), 40S, 60S/80S, and polysomes (Fig. 3H). Equal amounts of each fraction were analyzed by western blotting and RPS6 and RPL26 were used to monitor the 40S and 60S particles, respectively. EWSR1 was mostly present in fractions corresponding to the free pool of mRNA but was also detected in the 40S fractions. In contrast, it was never observed in the 60S/80S or polysome fractions. Next, we sought to assess the association of EWSR1 with messenger ribonucleoprotein (mRNP) complexes. To this aim, we irradiated HeLa cells with UV to covalently cross-link direct RNA–protein interactions and then pulled down poly(A) mRNPs from both total and cytoplasmic extracts using olido(dT)-tagged beads. RBPMS, a well characterized RBP [89], and GAPDH and Histone H3, both of which have no known mRNP-related function were used as positive and negative controls, respectively. EWSR1 was efficiently pulled down from both the total and cytoplasmic fractions, in an RNA-dependent manner (Fig. 3I). Together with the results of our polysome profiling, these observations are consistent with a previous study reporting that EWSR1 was highly enriched in small, ribosome-free mRNP complexes [90]. We thus concluded that EWSR1 is mostly associated with non-translating mRNAs.

### EWSR1 interacts with the 40S particle via its RGG2–ZnF–RGG3 region

Our observations that EWSR1 co-sediments with 40S on polysome gradient led us to hypothesize that EWSR1 might inhibit translation by interacting with the small ribosomal subunit. To test this hypothesis, we first performed co-immunoprecipitations between FLAG-tagged EWSR1 or its isolated NTD or CTD regions and endogenous components of the 40S and 60S ribosomal particles from HeLa cells. We found that both EWSR1 and its CTD associated with components of the 40S (i.e. RPS5 and RPS6) and 60S (i.e. RPL22 and RPL26) particles (Fig. 4A). In contrast, the NTD region of EWSR1 showed no association with the tested ribosomal proteins, indicating that the association between ribosomal components and EWSR1 is mediated solely by its CTD. Intriguingly, among FET members, the ability to immunoprecipitate with ribosomal components was observed for EWSR1 and FUS, but not TAF15 (Supplementary Fig. S4A). Based on this observation, we tested FUS and TAF15 in our translation reporter assay. Surprisingly, we observed that both FUS and TAF15 inhibited the translation of the *RLuc-4MS2* reporter mRNA (Supplementary Fig. S4B–D). To discriminate between potential indirect interactions mediated by mRNAs and mRNA-independent interactions, we repeated the co-immunoprecipitations between EWSR1 and 40S and 60S components in the presence of RNase A. RNase A has been shown to digest mRNA while preserving the integrity of 80S particles, as confirmed by co-immunoprecipitations between FLAG–RPL22 and endogenous components of the 40S particle (Supplementary Fig. S4E) [91]. The levels of 60S components RPL22 and RPL26 co-immunoprecipitating with EWSR1 or its CTD were significantly reduced under RNase A treatment, while association with the 40S components RPS6 and RPS23 remained largely unaffected (Fig. 4B). Similar observations were repeated using RNase T1, which digests polyribosomes into monosomes (Supplementary Fig. S4F) [91]. Again, we found that the association between EWSR1 or its CTD with the 40S ribosomal protein RPS6 was largely RNase T1-insensitive (Supplementary Fig. S4G). Finally, binding of EWSR1 to the 40S particle was confirmed at the endogenous level (Fig. 4C). Altogether, these results demonstrate that EWSR1 interacts with the 40S particle, largely in a mRNA-independent manner.

**Figure 4.**
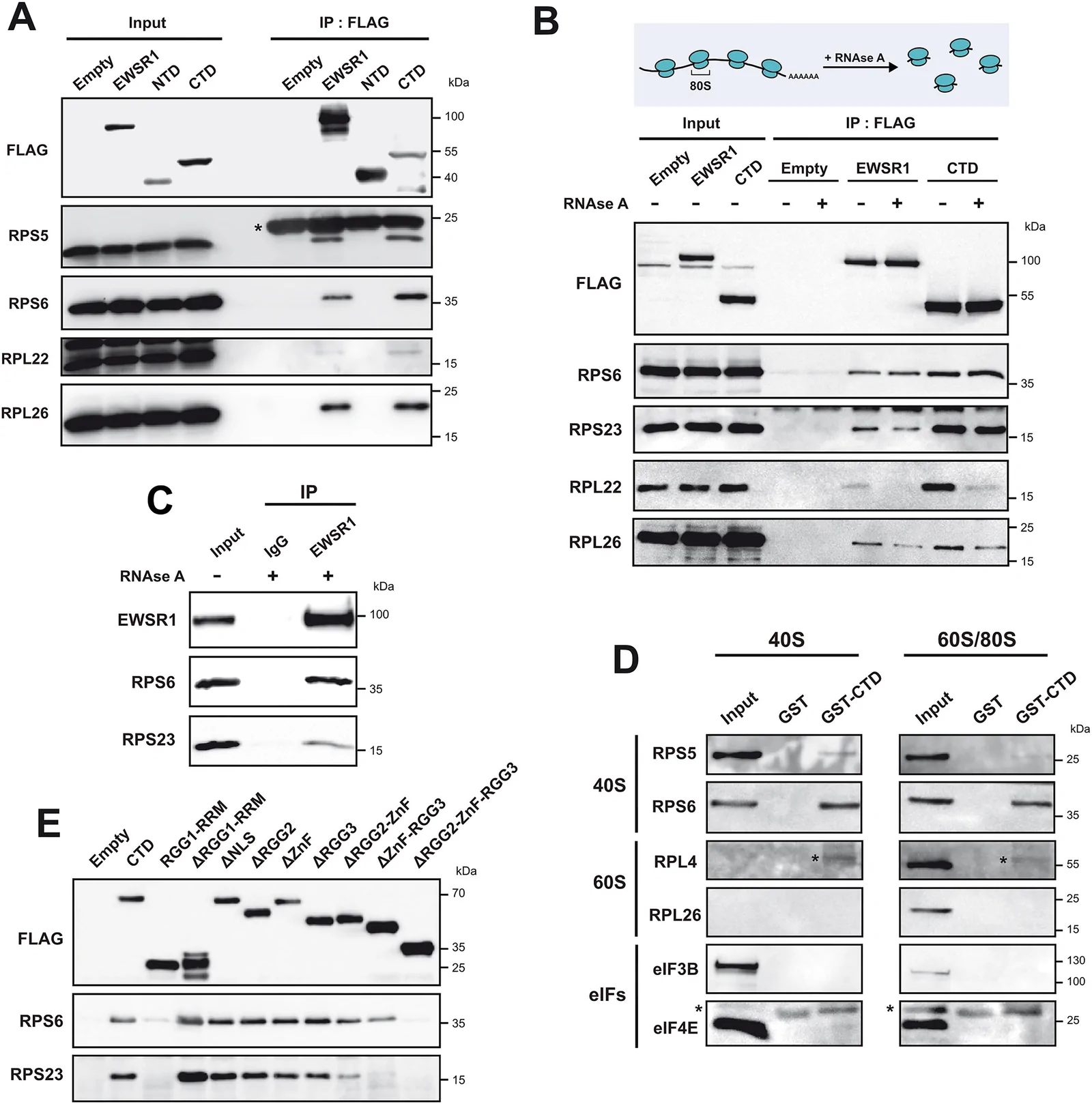
EWSR1 interacts with the 40S particle via its RGG2–ZnF–RGG3 region. (**A**) Western blot analysis of FLAG immunoprecipitates (IP) from HeLa cells transfected with FLAG–EWSR1, FLAG-NTD, FLAG-CTD, or control FLAG Empty-vector using the indicated antibodies. RPS5 and RPS6 are used as representative components of 40S, while RPL22 and RPL26 are used as representative components of 60S. Asterisk indicates a non-specific band. (**B**) (Top) Schematic representation of RNase A treatment effect on polysomes. (Bottom) Western blot analysis of FLAG immunoprecipitates (IP) from HeLa cells transfected with FLAG–EWSR1, FLAG-CTD, or control FLAG Empty-vector using the indicated antibodies. RPS6 and RPS23 are used as representative components of 40S, while RPL22 and RPL26 are used as representative components of 60S. Cell lysates were treated (+) or not (−) with RNase A prior to immunoprecipitation. (**C**) Immunoprecipitation (IP) of endogenous EWSR1 from HEK293 cell lysates followed by western blotting for the indicated proteins. RPS6 and RPS23 are used as representative components of 40S. (**D**) Western blot analysis of GST pulldowns performed with equal amount of recombinant GST-CTD or GST alone. Pulldowns were done on 40S or 60S/80S fractions (Input) from sucrose density fractionation of HeLa cytoplasmic extracts. Purity of the 40S and 60S/80S fractions was assessed using specific antibodies against various 40S and 60S components and initiation factors (IFs). Equal levels of GST-tagged proteins in the pulldown reactions are shown in Supplementary Fig. S4H. Asterisks indicate nonspecific bands. (**E**) Western blot analysis of FLAG immunoprecipitates (IP) from HeLa cells transfected with the FLAG Empty-vector or constructs encoding FLAG-tagged versions of wild-type EWSR1 CTD or the indicated deletion mutants. RPS6 and RPS23 are used as representative components of 40S.

To further characterize the association between EWSR1 and the 40S particle, we performed GST pulldown assays with a purified CTD fragment (GST-CTD) produced in *E. coli* as a GST fusion protein (GST-CTD). We incubated GST-CTD with 40S or 60S/80S fractions purified from polysome gradients. As expected, the purified 40S fraction contained 40S ribosomal proteins (RPS6 and RPS5) and initiation factors (eIF3B, eIF3η, and eIF4E), but no detectable 60S ribosomal proteins (RPL26 or RPL4) (see the “Input” lanes in Fig. 4D). In contrast, the 60S/80S fraction contained both 40S and 60S ribosomal proteins, but very little to no initiation factors. The 40S and 60S/80S fractions were independently incubated with comparable amounts of GST or GST-CTD (Supplementary Fig. S4H). Western blot analysis of pulled down proteins revealed specific association of the CTD with 40S ribosomal proteins, such as RPS6 and RPS5 (Fig. 4D). In contrast, no association was observed between GST-CTD and tested 60S components, supporting an association of the EWSR1 CTD with the 40S particle in the absence of an associated 60S subunit. Because the initiation factors present in the 40S fractions, such as the cap-binding eIF4E, or the 43S pre-initiation factor eIF3 were not pulled down by GST-CTD, these experiments further argue against association of EWSR1 with a canonical 43S PIC.

These observations prompted us to better map the 40S-interacting region of EWSR1 within the CTD. To this aim, we tested the ability of our CTD deletion mutants (see Fig. 2G) to co-immunoprecipitate with endogenous 40S ribosomal proteins RPS6 and RPS23. These experiments identified two mutants that were unable to associate with the 40S particle: RGG1–RRM, which lacks the RGG2–ZnF–RGG3–NLS region and the ΔRGG2-ZnF-RGG3 mutants (Fig. 4E and Supplementary Fig. S4I). Although the ΔRGG2-ZnF and ΔZnF-RGG3 mutants were severely impaired in their ability to associate with RPS6 and RPS23, they retained some binding. The mutants lacking either the RGG2 or the RGG3 (i.e. ΔRGG2 or ΔRGG3 respectively) were not significantly affected. These results thus identify the RGG2–ZnF–RGG3 region of EWSR1 as the minimal region responsible for the interaction with the 40S particle. Indeed, an EWSR1 mutant lacking the RGG2–ZnF–RGG3 region failed to associate with RPS6, demonstrating that no regions, potentially located in the NTD of EWSR1 contribute to this interaction (Supplementary Fig. S4J). Strikingly, this same RGG2–ZnF–RGG3 region was also identified as being involved in EWSR1-mediated translational repression. The overlap between the region contributing to 40S association and the region required for translation repression indicates that these two activities are functionally associated, although the present experiments do not establish whether 40S binding contributes directly to repression.

### EWSR1 regulates endoplasmic reticulum homeostasis

Next, we sought to evaluate the biological relevance of the translational function of EWSR1. To this aim, we performed Gene Ontology (GO) term enrichment analysis of the 595 TE up genes (see Fig. 1C). EWSR1 translational targets were functionally related to multiple aspects of the endoplasmic reticulum (ER), such as ER membranes [“*protein localization to endoplasmic reticulum*” (GO:0070972), “*endoplasmic reticulum lumen*” (GO:0005788) or “*lysosomal lumen*” (GO:0043202)], glycosylation [*e.g*. “*Extracellular matrix organization”* (GO:0030198), “*Glycosaminoglycan metabolic process*” (GO:0030203) or “*Glycoprotein metabolic process*” (GO:0009100) or “*Protein glycosylation*” (GO:0006486)], or lipid metabolism [e.g. “*membrane lipid metabolic process*” (GO:0006643), “*sphingolipid metabolic process*” (GO:0006665)] (Fig. 5A). GO analysis of the 132 “putative EWSR1 translational targets” (see Fig. 2A) identified processes similar to those of the TE up genes (Supplementary Fig. S5A). In contrast, the 222 TE down genes were enriched in processes linked to gene expression, such as RNA processing and translation (Supplementary Fig. S5B). Altogether, these analyses showed that EWSR1 translational targets are specifically linked to ER-associated processes and proteins.

**Figure 5.**
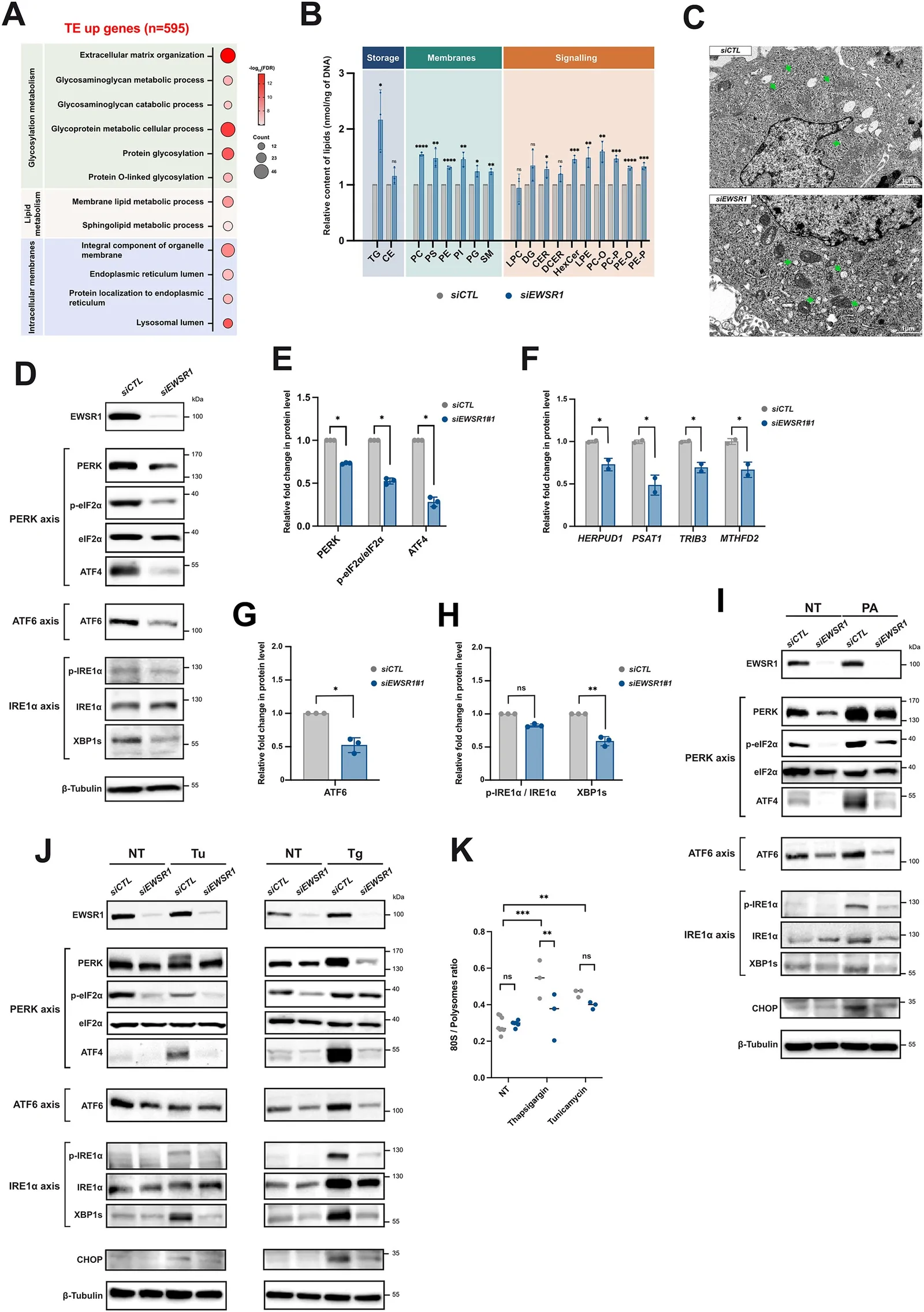
The translational function of EWSR1 is linked to lipid homeostasis. (**A**) Bubble charts of Gene Ontology (GO) Biological Process terms in TE up mRNAs (FDR < 0.05). Bubble’s size reflects the number of DTEGs and color scale corresponds to *P*-values, as indicated in the scales. *P*-values were calculated using FDR correction. (**B**) Fold change in *siEWSR1*-treated versus *siCTL*-treated HeLa cells (48 h siRNA treatment) of the classes of lipids described in Supplementary Fig. S5E. Results are means ± SD from three independent experiments. *P < 0.05, **P < 0.01; ***P < 0.001, ^****^P < 0.0001, ns = not significant compared to the *siCTL* condition by two-tailed unpaired Student’s *t*-test. (**C**) TEM images showing ER changes in HeLa cells transfected with *siCTL* or *siEWSR1#1* for 48 h. Green arrows points to ER structures; scale bars: 1 μm. (**D**) Representative western blot analysis of EWSR1 and components of the PERK, ATF6, and IRE1α branches of the UPR^ER^ in total cell lysates from HeLa cells transfected with *siCTL* or *siEWSR1#1* for 48h. β-Tubulin is used as a loading control. (**E**) Protein levels quantification of PERK, p-eIF2α, eIF2α, and ATF4 after western blotting and band densitometry analysis in total cell lysates from HeLa cells transfected with *siEWSR1#1* or *siCTL* for 48 h. Values were normalized to β-tubulin. Data are shown as means ± SD from three independent experiments, expressed relative to *siCTL* condition. Statistical analysis was performed using two-way repeated-measures ANOVA followed by Šídák’s multiple-comparisons test comparing *siCTL* and *siEWSR1* for each marker; *P < 0.05. (**F**) Fold change in mRNA levels of the four downstream targets of ATF4 (*HERPUD1, PSAT1, TRIB3*, and *MTHFD2*) in *siCTL* and *siEWSR*1-transfected Hela cells retrieved from the RNA-seq data described in Supplementary Fig. S1E. *P < 0.05 compared to the *siCTL* condition by multiple *t-*test. (**G, H**) Quantification of ATF6 protein levels (G) and of IRE1α phosphorylation and XBP1s expression (H) after western blotting as described in Fig. 5D and band densitometry analysis in total cell lysates from HeLa cells transfected with *siEWSR1#1* or *siCTL* for 48 h. Data are shown as means ± SD from three independent experiments, expressed relative to *siCTL* condition. Statistical analysis was performed on raw quantification values normalized to β-tubulin using two-way repeated-measures ANOVA followed by Šídák’s multiple-comparisons test comparing *siCTL* and *siEWSR1* for each marker; *P < 0.05, **P < 0.01, ns = not significant. (**I**) Representative western blot analysis of EWSR1 and components and distal downstream targets CHOP of the PERK, ATF6, and IRE1α branches of the UPR^ER^ in total cell lysates from HeLa cells transfected with *siCTL* or *siEWSR1#1* and treated (PA) or not (NT) by palmitic acid. β-Tubulin is used as a loading control. (**J**) Representative western blot analysis of EWSR1 and components and distal downstream targets (CHOP and P21) of the PERK, ATF6 and IRE1α branches of the UPR^ER^ in total cell lysates from HeLa cells transfected with *siCTL* or *siEWSR1#1* for 48 h and treated or not (NT) by tunicamycin (Tu) for 5 h or thapsigargin (Tg) for 24 h. β-Tubulin is used as a loading control. (**K**) HeLa cells transfected with *siCTRL* or *siEWSR1* for 48 h were left untreated (NT), treated with thapsigargin for 24 h, or treated with tunicamycin for 5 h prior to polysome profiling analysis. The graph shows the ratio between the 80S monosome area and the polysome area calculated from polysome profiles obtained in at least three independent biological experiments. Statistical analyses were performed using mixed-effect Model Anova with Šídák’s multiple comparisons test. ns = not significant; **P < 0.01; ***P < 0.001.

Many TE up genes were linked to lipid metabolism, which mainly takes place in the ER. In addition, fluctuations in lipid homeostasis translate into changes in ER shape and function [92]. This connection was specific to the translational function of EWSR1, as no lipid-related GO was enriched in DEG (see Supplementary Fig. S1F). We thus hypothesized that EWSR1 might control cellular lipid composition via its translational function. To support this idea, we first tested the ability of EWSR1 to bind transcripts related to lipid metabolism. Among the 132 “putative EWSR1 translational targets,” we selected 8 transcripts encoding key regulators of lipid metabolism (*FADS1, FADS2, ELOVL5, ELOVL6, LPCAT1, LAMP1, SLC39 A1, HSD17B12*) for validation by RIP-qPCR. EWSR1 binding was confirmed for the majority of these candidates (5/8), whereas *ELOVL5, FADS1 and LPCAT* did not exhibit significant enrichment in the assay (Supplementary Fig. S5C). Next, we conducted a lipidomic analysis in EWSR1–KD cells using mass spectrometry (Supplementary Fig. S5D,E). Except for cholesterol esters (CE), lysophosphatidylcholine (LPC), diacylglycerides (DG), and dihydroceramides (DCER), all lipid classes were significantly increased in EWSR1–KD cells (Fig. 5B). Altogether, these observations suggest that EWSR1 negatively regulates cellular lipid production via its translational function.

Because EWSR1 translational targets were associated with processes linked to ER homeostasis, we first assessed the morphology of ER in EWSR1–KD cells using transmission electron microscopy. We found that EWSR1–KD cells had an altered ER, appearing as dilated structures with expanded lumen (Fig. 5C). In contrast, no morphological difference was detected for the nucleus, Golgi, lysosomes, or mitochondria between control and EWSR1-depleted conditions (Supplementary Fig. S5F). ER perturbations in protein and lipid homeostasis trigger an adaptive stress response known as the unfolded protein response (UPR^ER^) [93]. We thus investigated the UPR^ER^ status of EWSR1–KD cells, using classical markers of the three UPR^ER^ branches. We found that KD of EWSR1 was associated with a significant reduction in PERK levels (Fig. 5D and E). In addition, we detected an almost 2-fold reduction (i.e. 46%) in the phosphorylation of its downstream target eIF2α and an almost 4-fold (i.e. 76%) decrease in ATF4 protein levels (Fig. 5D and E). Finally, classical ATF4 targets, such as *HERPUD1, PSAT1, TRIB3*, and *MTHFD2*, were downregulated in EWSR1–KD cells (Fig. 5F). These observations demonstrated that activation of the PERK/ATF4 branch was reduced in EWSR1–KD cells. In parallel, we also observed that KD of EWSR1 correlated with an inhibition of the ATF6 branch of the UPR^ER^, as assessed by a decrease in ATF6 levels (Fig. 5D and G). Finally, EWSR1–KD cells exhibited reduced IRE1α phosphorylation, although this effect did not reach statistical significance, whereas *XBP1* mRNA splicing was significantly decreased, indicating attenuation of the IRE1α axis (Fig. 5D,H and Supplementary Fig. S5G). Importantly, these effects were validated with a second siRNA against EWSR1 (i.e. *siEWSR1#2*) (Supplementary Fig. S5H and I) and in two other unrelated human cancer cell lines (A2780A and U2OS) (Supplementary Fig. S5J). These observations thus showed that depletion of EWSR1 mitigates constitutive ER stress and reduces basal UPR^ER^ activation in cancer cells.

Based on our findings, we next sought to assess the role of EWSR1 in stress-induced UPR^ER^. To this aim, we first exposed control and EWSR1–KD HeLa cells to lipid stress by palmitic acid (PA) and monitored activation of the UPR^ER^. As expected, exposure to PA powerfully activated the UPR^ER^ in EWSR1-expressing cells (Fig. 5I). However, activation of the three branches of UPR^ER^ by PA was dramatically reduced in EWSR1–KD cells. C/EBP homologous protein (CHOP), also known as growth arrest and DNA damage-inducible protein 153 (DDIT3/GADD153) is a stress-responsive transcription factor onto which converge all three branches of the mammalian UPR^ER^. Using expression of CHOP as a proxy for distal activation of the UPR^ER^, we found that CHOP expression was systematically reduced in EWSR1–KD, both in nontreated (NT) or in PA-treated cells. Interestingly, KD of EWSR1 also reduced activation of the UPR^ER^ by proteotoxic stress caused by tunicamycin (Tu) or thapsigargin (Tg) (Fig. 5J). To determine whether the impaired UPR activation observed in EWSR1-depleted cells was associated with altered translational remodeling, we performed polysome profiling analyses following thapsigargin or tunicamycin treatment. Quantification of the 80S-to-polysome ratio revealed that EWSR1 depletion had no significant effect under basal conditions, consistent with the polysome profiles and Sunset assays shown in Figure 1. In contrast, both thapsigargin and tunicamycin induced a significant increase in the 80S/polysome ratio in control cells, whereas this response was attenuated following EWSR1 depletion (Fig. 5K). Altogether, these observations demonstrate that EWSR1 is necessary for full activation of proximal and distal UPR^ER^ signaling.

## Discussion

The FET protein family exemplifies the multifunctionality of RBPs, as its members have been shown to control transcription, mRNA processing, and cytoplasmic fates of mRNA, although this latter aspect is much less characterized [34]. Despite its high pathological relevance and involvement in multiple oncogenic fusions [34, 94], the normal physiological functions of the FET member EWSR1 remain paradoxically fragmentary. Previous studies reporting the association of EWSR1 with ribosomes [95], its presence in SGs [29] and its potential role in post-transcriptional processes of gene expression [96, 97] led us to investigate its role in translation. Here, we provide evidence that EWSR1 functions as a selective translational repressor. Specifically, we show that EWSR1 inhibits translation upon association with target transcripts and that a region of its CTD, spanning the RGG2–ZnF–RGG3 domains, associates with the 40S ribosomal subunit. Furthermore, we demonstrate that EWSR1-mediated regulation of mRNA translation impacts a specific set of mRNAs linked to ER-related processes, such as lipid homeostasis. We show that EWSR1 is essential for ER homeostasis in cancer cells. Indeed, absence of EWSR1 impacts ER morphology and impairs UPR^ER^ activation. These results provide new insights into the physiological role of EWSR1 as a key regulator of UPR^ER^ by modulating specific mRNA translation events. Taken together, our study advances the understanding of EWSR1 function in gene regulation and highlights its broader implications in cellular metabolism and stress responses.

Although our study focuses on EWSR1, an interesting question is whether FUS and TAF15 also share the ability to repress translation. Using a translation reporter assay, we found that tethering FUS or TAF15 to the 3′UTR of an RLuc reporter transcript significantly reduced luciferase mRNA translation (Supplementary Fig. S4B–D). This suggests that all FET family members share the capacity for inhibiting translation. Our results align with previous studies implicating ALS-associated FUS mutants in translational repression. However, these studies often overlooked the role of wild-type FUS in this process [30–32, 33, 98]. To our knowledge, our study provides the first direct evidence of TAF15 involvement in translational regulation, though further investigation is warranted. Notably, we observed that TAF15 did not interact with ribosomal components (Supplementary Fig. S4A), suggesting it may repress translation through a mechanism distinct from EWSR1 and FUS. Potential mechanisms include TAF15 binding to 3′UTR [99], its participation in translation regulatory modules [100] or its involvement in the miRNA machinery [101]. At first glance, the inability of TAF15 to associate with ribosomal components may seem surprising, given the high sequence similarity among FET family members. However, the CTD of TAF15 is the most divergent within the family. Notably, its RGG3 region consists almost exclusively of YGGDRG/SG repeats, which are completely absent in EWSR1 and FUS [8]. To date, the mechanistic and structural determinants underlying TAF15-mediated translational repression remain to be fully elucidated.

Transcriptome-wide RNA-binding analyses have revealed that EWSR1 binds thousands of transcripts [20, 84]. However, efforts to define the RNA-binding modes of EWSR1 have yet to identify a clear canonical binding motif, aside from a preference for G-rich sequences and motifs [83, 84]. The current consensus is that EWSR1 recognizes specific RNA secondary structures, such as stem-loops or G-rich sequences folded into rG4 [10, 26, 65]. Notably, our analysis revealed an enrichment of rG4 structures in EWSR1 translational targets, especially within their 3′UTR (Fig. 1I), suggesting that these structures may serve as key determinants for EWSR1 binding to specific mRNAs. Additionally, rG4 are known to hinder cap-dependent and IRES-mediated translation through various mechanisms [102]. Thus, it is possible that EWSR1 recruitment to rG4-containing transcripts facilitates the folding or stabilizing of these structures, thereby contributing to translational repression. Supporting this model, several rG4-binding proteins are established translational regulators, including DDX3X, Aven, Nucleolin, CCHC-type zinc finger nucleic acid-binding protein (CNBP/ZNF9), or Fragile X Syndrome ribonucleoprotein (FMRP) [103, 104, 105].

Through detailed mutational analysis, we identified the RGG2–ZnF–RGG3 region of EWSR1 as critical for its interaction with the 40S ribosomal particle and for its translational repressive activity. Although these findings remain correlative, they align with previous observations that RGG motifs in translational repressors play a key role in binding ribosomes, a crucial aspect of their repressive function [6, 106, 107]. Further efforts to map the 40S-interaction surface within EWSR1 yielded intriguing results (Fig. 4E). Specifically, mutants lacking only the RGG2, ZnF, or RGG3 domains retained their association with 40S. However, mutants lacking the RGG2–ZnF or ZnF–RGG3 regions exhibited a distinct binding pattern: while they maintained a strong interaction with RPS6, their association with RPS23 was markedly reduced. These findings suggest that EWSR1 binds to the 40S ribosomal subunit through a complex, multimodal mechanism, with individual domains within the RGG2–ZnF–RGG3 region contributing to interactions with distinct 40S components. Although our results demonstrate that the RGG2–ZnF–RGG3 region is required for EWSR1-mediated translational repression, we cannot rule out contributions from other regions. For example, the RRM and RGG1 domains may assist in mRNA binding and determining translational target specificity, while the PrLD could promote the formation of higher-order molecular assemblies that may contribute to EWSR1 function [108].

How does EWSR1 regulate translation? Multiple lines of evidence suggest that EWSR1-mediated translational repression involves association with specific mRNAs. Both in RRL and *in vivo* translation reporter assays, tethering EWSR1 to mRNA was necessary for effective translational repression of the reporter mRNA. This indicates that EWSR1 is not a general inhibitor of translation but rather exerts its repressive effects when recruited to specific mRNAs. Indeed, the set of translation targets identified in this study is enriched with features known to promote EWSR1 binding and includes many previously identified EWSR1-bound mRNAs. Together, these findings support a model in which EWSR1 inhibits translation through association with specific target mRNAs, although we cannot fully exclude the possibility that indirect effects may also contribute. The RGG2–ZnF–RGG3 region contributes both to translational repression and to association with 40S-containing complexes, suggesting that these activities are functionally linked. However, because this region may also contribute to RNA binding and/or protein-protein interactions, our experiments do not establish that 40S association itself is the causal determinant of translational repression.

Our experiments further provide constraints on the translational step affected by EWSR1. EWSR1 or its CTD repress translation from PV-IRES, CrPV-IRES, and TISU-driven reporters, indicating that repression does not require canonical cap recognition or 43S scanning. In addition, the EWSR1 CTD specifically associates with 40S particles devoid of detectable initiation factors arguing against a mechanism involving canonical PICs. Although EWSR1 is reproducibly detected in 40S-containing fractions, it is mostly absent from 60S/80S and polysome fractions and does not behave as a constitutive ribosomal component. These observations are consistent with a model in which EWSR1 transiently associates with 40S-containing complexes in the context of translational repression. One possibility is that EWSR1 affects a late step of translation initiation, such as productive 60S joining or AUG recognition, although the precise molecular step cannot be resolved by the present experiments. Further studies will be required to determine how EWSR1-containing complexes engage target mRNAs and how this association alters productive translation.

EWSR1 is also known to participate in cytoplasmic ribonucleoprotein assemblies, and the punctate cytoplasmic distribution observed in our experiments raises the possibility that such assemblies contribute to its cellular function. Our fractionation experiments show that EWSR1 is predominantly associated with the free mRNA/mRNP pool, with a smaller fraction detected in 40S-containing fractions and little to no detectable association with 60S/80S or polysomal fractions. Thus, the possibility that EWSR1 participates in mRNA sequestration or remodeling within cytoplasmic RNP assemblies cannot be completely excluded. However, the translational repressive activity of purified EWSR1 CTD in a cell-free translation system indicates that cellular compartmentalization or formation of cytoplasmic condensates is not required for EWSR1-mediated translational inhibition. These observations therefore support an intrinsic translational activity of EWSR1 while leaving open the possibility that RNP assemblies modulate this activity in cells.

An important remaining question is how and when EWSR1 associates with its translational target transcripts. EWSR1 is predominantly nuclear, consistent with its established roles in nuclear RNA processing. It is therefore possible that association with its translational targets occurs during transcription, mRNA processing or export, prior to their entry into the cytoplasm. Alternatively, EWSR1 may associate with target mRNAs after their export. The present experiments do not distinguish between these possibilities.

FET proteins have garnered significant interest due to their involvement in various neurological disorders and cancers. In neurodegenerative diseases, such as FTLD and ALS, pathogenic mutations in FUS often lead to its mislocalization into cytoplasmic aggregates [16]. Interestingly, two important ALS–FUS variants with mutations in the NLS region (i.e. R521G and P525L) associate with the 40S, 60S and 80S polysomal fractions and efficiently repress translation [31]. However, when mutated in their RGG2 domain, these ALS variants failed to inhibit translation, consistent with our observations highlighting the importance of the RGG2–ZnF–RGG3 region in EWSR1-mediated translational repression. Many ALS/FTLD mutations in FUS and EWSR1 fall within this region. It is thus essential to assess the impact of these mutations on the ability of FUS and EWSR1 to associate with polysomes and repress translation. EWSR1 is best known for its involvement in chromosomal translocations that produce oncogenic fusion proteins, which are pathognomonic for a wide range of soft tissues neoplasms. These fusions typically link the N-terminal PrLD region of EWSR1 to the DNA-binding domain of a transcription factor, as exemplified in EWSR1::FLI1, the driver of Ewing sarcoma [34]. Our findings suggest that the loss of or mutations in one FET allele may cause haploinsufficiency in EWSR1-mediated translation regulation, contributing to cancer development, a concept supported by growing evidence that translational dysregulation is a key factor in the pathogenesis of cancer [109]. Interestingly, Ewing sarcoma cell lines depend on high translational activity and are particularly sensitive to translation-inhibiting drugs [110, 111]. Based on our findings, we speculate that loss of one *EWSR1* allele could promote sarcomagenesis by increasing cellular translational activity. Additionally, Ewing sarcoma cells have been shown to rely on active biosynthesis of fatty-acids, sphingolipids and glycerophospholipids and are sensitive to lipid metabolism inhibitors [112]. While this dysregulated lipid metabolism has been attributed to EWSR1::FLI1-driven transcriptional activation of key lipid biosynthesis enzymes, our findings suggest that *EWSR1* haploinsufficiency may also contribute to lipid metabolism alterations in Ewing sarcoma cells, via increased translation of mRNA coding for lipid-related proteins. Overall, our work supports a model in which both EWSR1::FLI1 expression and EWSR1 haploinsufficiency jointly disturb normal cellular functions and coordinatively contribute to sarcomagenesis [14, 113]. This model, which may extend to other EWSR1-associated cancers, could pave the way for novel therapeutic strategies.

## Supplementary Material

gkag1022_Supplemental_Files

## Data Availability

Reagents generated in this study will be gladly made available upon request, but may require a payment for shipping and/or a completed Materials Transfer Agreement if there is potential for commercial application. RNA-seq and Ribo-Seq data have been deposited to NCBI SRA database, Bioproject PRJNA1278961.
