# Supplementary material for: EWSR1 associates with the 40S ribosomal subunit and represses translation to maintain endoplasmic reticulum homeostasis
Source: Nucleic Acids Res. 2026 Oct 29;54(20):gkag1022. doi: 10.1093/nar/gkag1022 (PMC13717198; doi:10.1093/nar/gkag1022)
Supplement: Supplementary file 1 [file gkag1022_supplemental_files.zip › Legends_Supplementary_Tables.pdf]

Supplementary Table S1. Differential gene expression analysis following EWSR1 depletion. DESeq2 output for the 2,282 genes differentially expressed between *siEWSR1*- and *siCTL*-transfected HeLa cells, including Ensembl gene IDs, mean normalized counts (baseMean), log2 fold changes, standard errors (lfcSE), Wald statistics, *P* values, adjusted *P*values (padj), HGNC gene symbols, and gene biotypes.

Supplementary Table S2. Differential translation efficiency analysis following EWSR1 depletion. Differential translation efficiency analysis output with DESeq2 for the 595 mRNAs exhibiting significantly increased translation efficiency in *siEWSR1*- compared with *siCTL*-transfected HeLa cells, including Ensembl gene IDs, mean normalized counts (baseMean), log2 fold changes, standard errors (lfcSE), Wald statistics, P values, adjusted P values (padj), HGNC gene symbols, and gene biotypes.

Supplementary Table S3. Differential pattern (DP) analysis of ribosome footprint distribution following EWSR1 depletion. List of the 1,213 mRNAs exhibiting significantly different ribosome-protected fragment (RPF) distribution patterns between siEWSR1- and siCTL-transfected HeLa cells, including Ensembl gene IDs, P values, and FDR-adjusted q values.

Supplementary Table S4. High-confidence EWSR1-bound transcript dataset. Compilation of 2,778 high-confidence EWSR1-bound mRNAs identified in three independent CLIP-seq studies (Hoell *et al*., 2011; Paronetto *et al*., 2014; Van Nostrand *et al*., 2020). The table includes Ensembl gene IDs, HGNC gene symbols, and indicates whether each transcript was identified in each of the three CLIP-seq datasets.
