# Supplementary material for: EWSR1 associates with the 40S ribosomal subunit and represses translation to maintain endoplasmic reticulum homeostasis
Source: Nucleic Acids Res. 2026 Oct 29;54(20):gkag1022. doi: 10.1093/nar/gkag1022 (PMC13717198; doi:10.1093/nar/gkag1022)
Supplement: Supplementary file 1 [file gkag1022_supplemental_files.zip › Supplementary Figures and legendsv2.pdf]

Supplementary Figures and legends

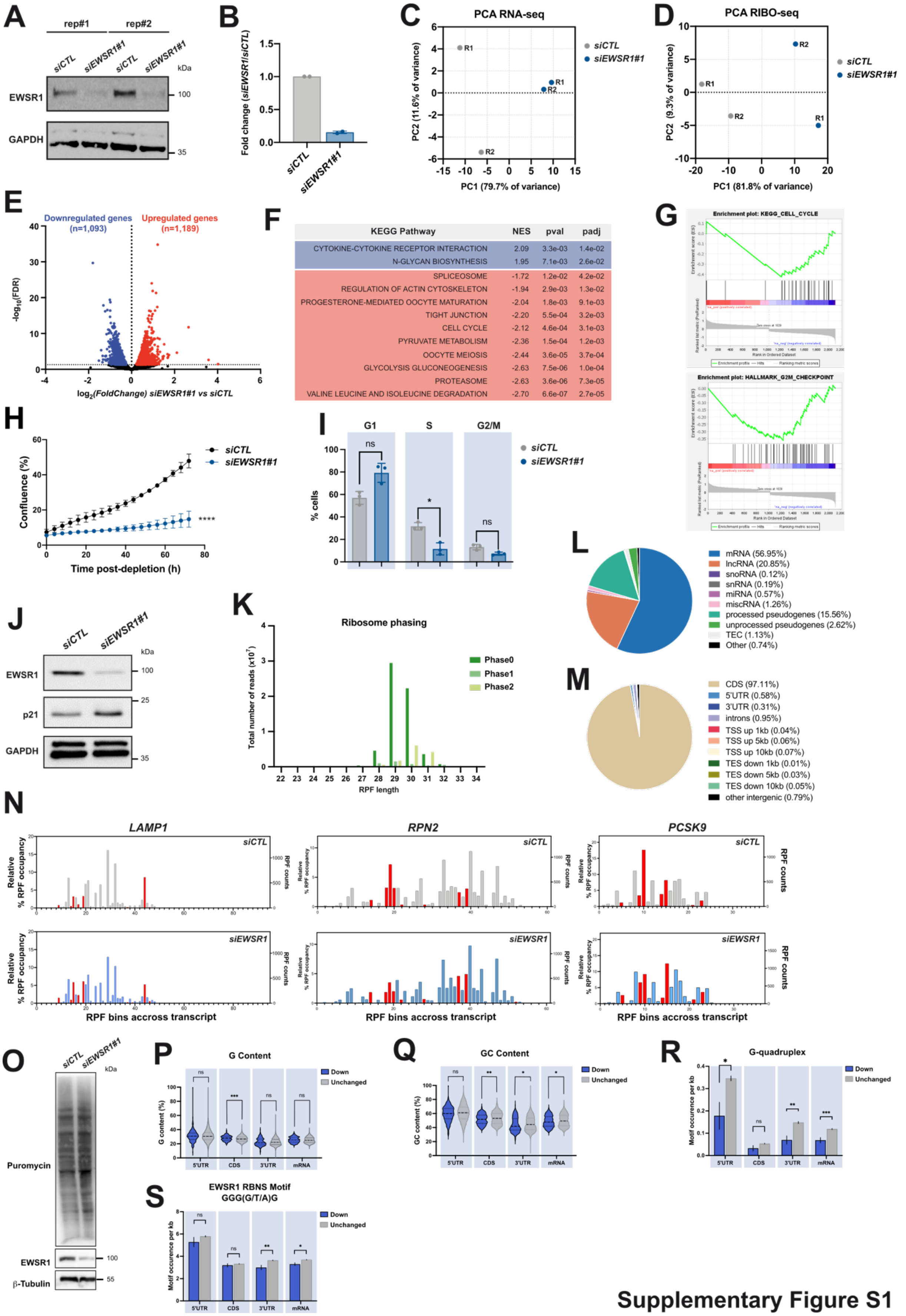

Supplementary Figure S1

Supplementary Figure S1. (**A**) Western blot analysis of EWSR1 and GAPDH (loading control) in total cell lysates from HeLa cells transfected with *siEWSR1#1* or *siCTL* for 48h and used in the Ribo-Seq analysis. (**B**) Quantification of EWSR1 signal from (**A**) by densitometry analysis using ImageJ. Results are shown as means ± SD (n = 2 independent experiments) after normalization to GAPDH and expressed as fold change relative to *siCTL* condition. (**C,D**) Principal Component Analysis (PCA) of RNA-seq (**C**) and Ribo-seq (**D**) libraries from *siCTL* and *siEWSR1#1* HeLa cells (n = 2 independent experiments). (**E**) Volcano plot of significantly downregulated (blue, n = 1,093) and upregulated (red, n = 1,189) genes upon EWSR1 knockdown in HeLa cells, as detected by RNA-seq analysis (n = 2 independent experiments). Black dots are genes with unchanged expression. Numbers refer to numbers of genes either up or downregulated. Horizontal dotted black line corresponds to FDR = 0.05. (**F**) Gene Set Enrichment Analysis (GSEA) of differentially expressed genes in EWSR1 KD cells. Signaling pathways are ranked based on normalized enrichment scores (NESs). Positive (blue) and negative (red) NESs indicate up- or down-regulation, respectively. (**G**) Enrichment plots from GSEA related to "*Cell cycle*" and "*G2M_Checkpoint*" downregulated in Hela cells upon KD of EWSR1. (**H**) Cell confluence measured using Incucyte live-cell imaging in HeLa cells treated for 48h with *siCTL* and *siEWSR1* showing that EWSR1 KD is associated with reduced proliferation. ****P<0.0001. (**I**) Percentage of HeLa cells in G1, S and G2/M phases of cell cycle 48h after transfection with *siCTL* or *siEWSR1#1*. Results are shown as means ± SD (n = 3 independent biological experiments). Statistical significance was assessed using a two-way repeated-measures ANOVA followed by Šídák's multiple-comparisons test. *P < 0.05; ns = not significant. (**J**) Western blotting of EWSR1, P21 and GAPDH (loading control). Samples are total cell lysates from HeLa cells transfected for 48h with *siEWSR1* or *siCTL*. (**K**) Plot of ribosomal phasing (phase 0, 1 and 2) against the ribosome protected fragment (RPF) length as detected in HeLa cells by RIBO-Seq analysis. (**L**) Distribution of Ribo-Seq reads according to gene biotype. Percentage of reads mapping to each gene biotype are indicated. (**M**) Distribution of Ribo-Seq reads according to various genomic regions. Percentage of reads mapping to each genomic region are indicated. (**N**) Examples of representative genes (*LAMP1*, *RPN2* and *PSCK9*) with differences in ribosome occupancy patterns. Plotted are absolute (right axis) and relative (%, left axis) P-site-mapped RFP binned counts along transcript in *siCTL* (grey) and *siEWSR*1 (blue) conditions. Positions with significant differences (adjusted p-value < 0.05) in

RPF bin pattern between conditions, as detected by RiboDiPA are highlighted in red. (**O**) Representative western blot of puromycin, EWSR1 and β-Tubulin (loading control) as performed in the Surface SEnsing of Translation (SUnSET) assay described in **Fig. 1D** and used for quantification in **Fig. 1E**. Samples are total cell lysates from HeLa cells transfected with *siCTL* or *siEWSR1#1* for 48h. (**P-S**) Features of EWSR1 TE up targets. Comparison of G content (**P**), GC content (**Q**), rG-quadruplex (**R**) and EWSR1 RBNS motif occurrences between unchanged and TE down mRNAs in the 3'UTR, 5'UTRs and CDS region, and across the entire sequence. *P < 0.05, **P < 0.01; ***P < 0.001, ns = not significant using Wilcoxon-Mann-Whitney test. Results are shown as ± SEM (n = 2 independent experiments).

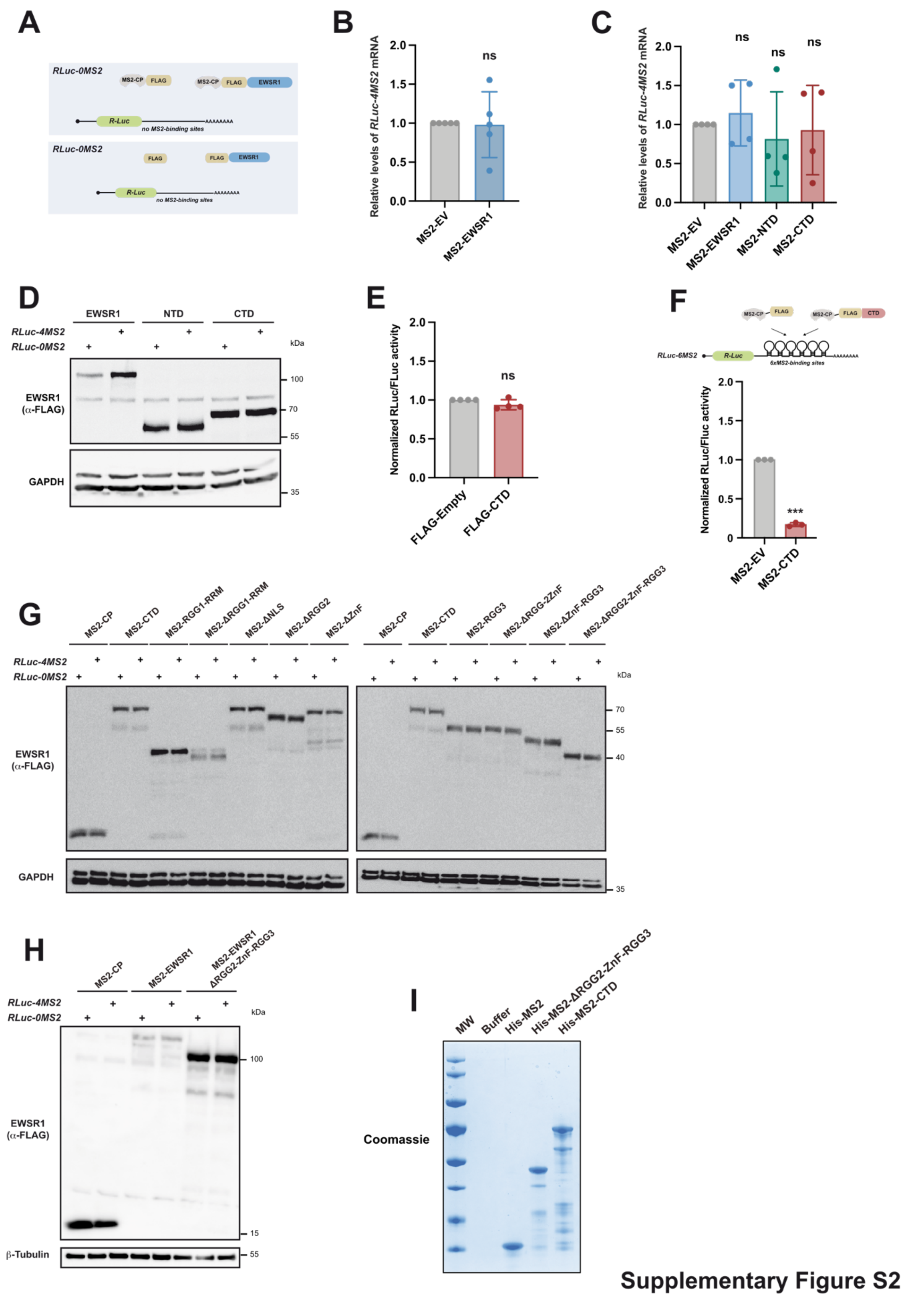

Supplementary Figure S2

Supplementary Figure S2. (**A**) Representation of the *RLuc-0MS2* reporter mRNA. Due to the lack of MS2-binding sites in its 3'UTR, this transcript does not allow tethering of MS2-CP-fused proteins. (**B**) Luciferase tethering assays in HeLa cells transfected with constructs coding for MS2-CP-tagged versions of FLAG-EV (MS2-EV) or FLAG-EWSR1 (MS2-EWSR1) together with the *RLuc-4MS2* or *RLuc-0MS2* reporters. Results are means of normalized RLuc-4MS2 mRNA levels [(*RLuc-4/FLuc*)/(*RLuc-0/FLuc*)], relative to the MS2-EV condition ± SD from five independent experiments. Statistical analyses were performed on the raw *RLuc-4MS2* mRNA levels using repeated-measures one-way ANOVA followed by Dunnett's multiple-comparisons test against EV, ns = not significant compared. (**C**) Luciferase tethering assays in HeLa cells transfected with constructs coding for MS2-CP-tagged versions of FLAG-EV (MS2-EV) or FLAG-tagged EWSR1 (MS2-EWSR1), NTD (MS2-NTD) or CTD (MS2-CTD) constructs together with the *RLuc-4MS2* or *RLuc-0MS2* reporters. Results are means of normalized *RLuc* mRNA levels, relative to the MS2-EV condition ± SD from four independent experiments. Statistical analyses were performed on the raw *RLuc*--4MS2 mRNA levels using repeated-measures one-way ANOVA followed by Dunnett's multiple-comparisons test against EV. ns: not significant. (**D**) Representative western blot analysis assessing comparable levels of MS2-EWSR1, MS2-NTD and MS2-CTD with an anti-FLAG antibody. GAPDH is used as a loading control. Samples are total cell lysates from HeLa cells used in (**C**). (**E**) Luciferase tethering assays in HeLa cells transfected with constructs coding for FLAG-EV (Empty vector) or FLAG-tagged EWRS1 CTD expressing constructs together with *RLuc-4MS2* or *RLuc-0MS2* reporters. Results are means of normalized *RLuc* mRNA levels, relative to the FLAG-EV condition ± SD from four independent experiments. ns: not significant compared to the MS2-Empty condition by one sample t test. (**F**) Representation of the *RLuc-6MS2* reporter mRNA (top). Luciferase tethering assays in HeLa cells transfected with constructs encoding MS2-CP-fused versions of FLAG-EV (Empty vector, MS2-EV) or FLAG-tagged CTD (MS2-CTD) expressing constructs together with *RLuc-6MS2* or *RLuc-0MS2* reporters (bottom). Results are means of normalized RLuc/FLuc activities [(RLuc-6/FLuc)/(RLuc-0/FLuc)] relative to the MS2-EV condition ± SD from three independent experiments). ***P <0.001 compared to the MS2-EV condition by one sample t test. (**G**) Representative western blot analysis assessing comparable expression levels of the indicated constructs with an anti-FLAG antibody. GAPDH is

used as a loading control. Samples are total cell lysates from HeLa cells used in **Fig. 2H**. (**H**) Representative western blot analysis of MS2-CP, MS2-EWSR1, MS2-NTD and MS2-CTD, with an anti-FLAG antibody. β-Tubulin is used as a loading control. Samples are total cell lysates from HeLa cells used in **Fig 2I**. (**I**) Coomassie staining assessing comparable levels of the recombinant His tag fused to MS2 (His-MS2) or His-tagged wild-type (HIS-MS2-CTD) or mutant (HIS-MS2-∆RGG2-ZnF-RGG3) EWSR1 CTD, as used in the *in vitro* translation reporter assay described in **Fig. 2L**.

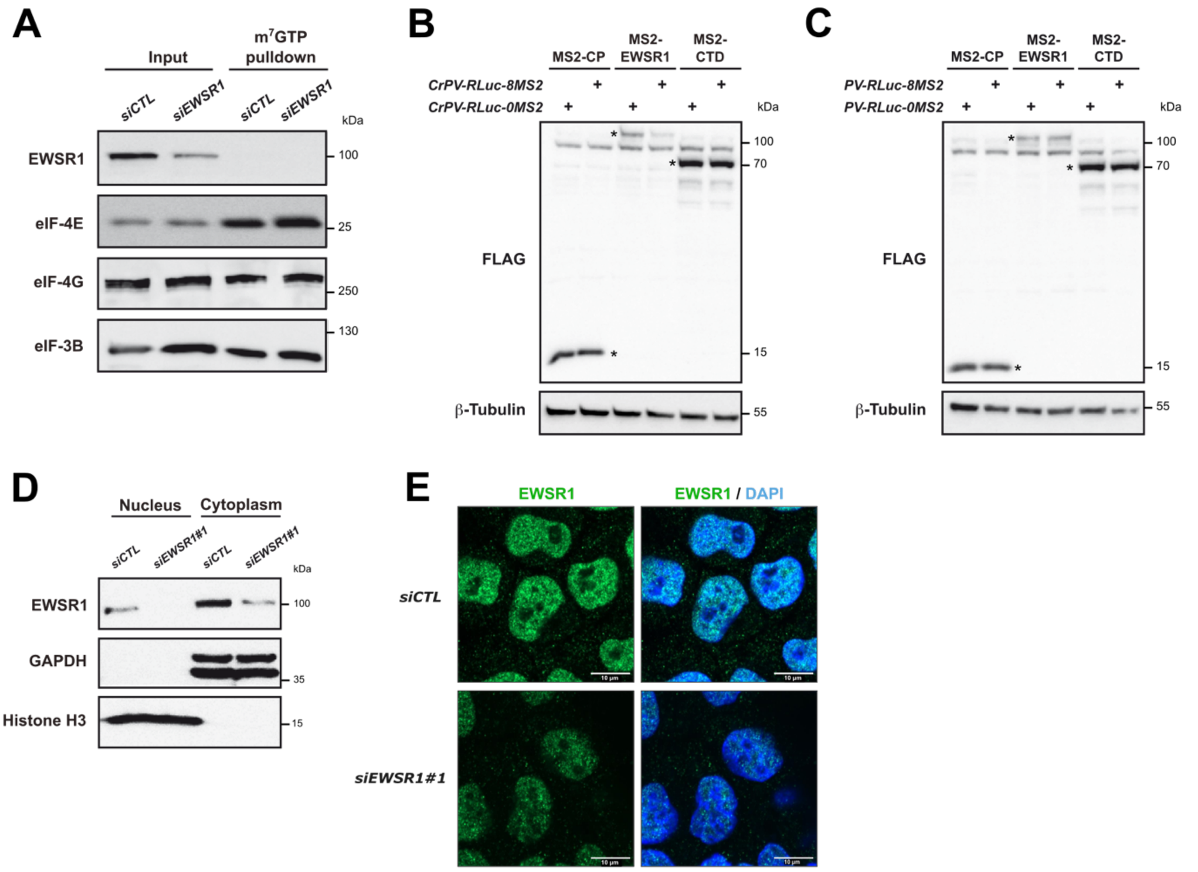

Supplementary Figure 3

Supplementary Figure S3. (**A**) Western blotting of EWSR1 and the indicated initiation factors in m^{7}GTP-agarose pulldowns from HeLa cells transfected for 48h with *siCTL* or *siEWSR1#1*. (**B,C**) Western blot analysis showing the expression of FLAG-tagged effector proteins used in the tethering assay shown in Fig. 3B. β-Tubulin is used as a loading control. Samples are total cell lysates from HeLa cells transfected with MS2-CP-FLAG (negative control) or MS2-CP-fused FLAG-EWSR1 or FLAG-CTD together with *CrPV-RLuc-8MS2* or *CrPV-RLuc-0MS2* (**B**) or *PV-RLuc-8MS2* or *PV-RLuc-0MS2* (**C**), as used in **Fig. 3B**. The position of MS2-CP, MS2-EWSR1 and MS2-CTD detected by anti-FLAG immunoblotting are indicated using asterix. (**D**) Western blotting of nuclear and cytoplasmic fractions from HeLa cells transfected with *siCTL* and *siEWSR1#1* for 48h. GAPDH and Histone H3 are used as control to assess the quality of the cytoplasmic and nuclear fractions, respectively. (**E**) Confocal immunofluorescence images of EWSR1 (green) and DAPI (blue) in HeLa cells transfected with *siCTL* and *siEWSR1*#1 for 48h. Scale = 10 µM.

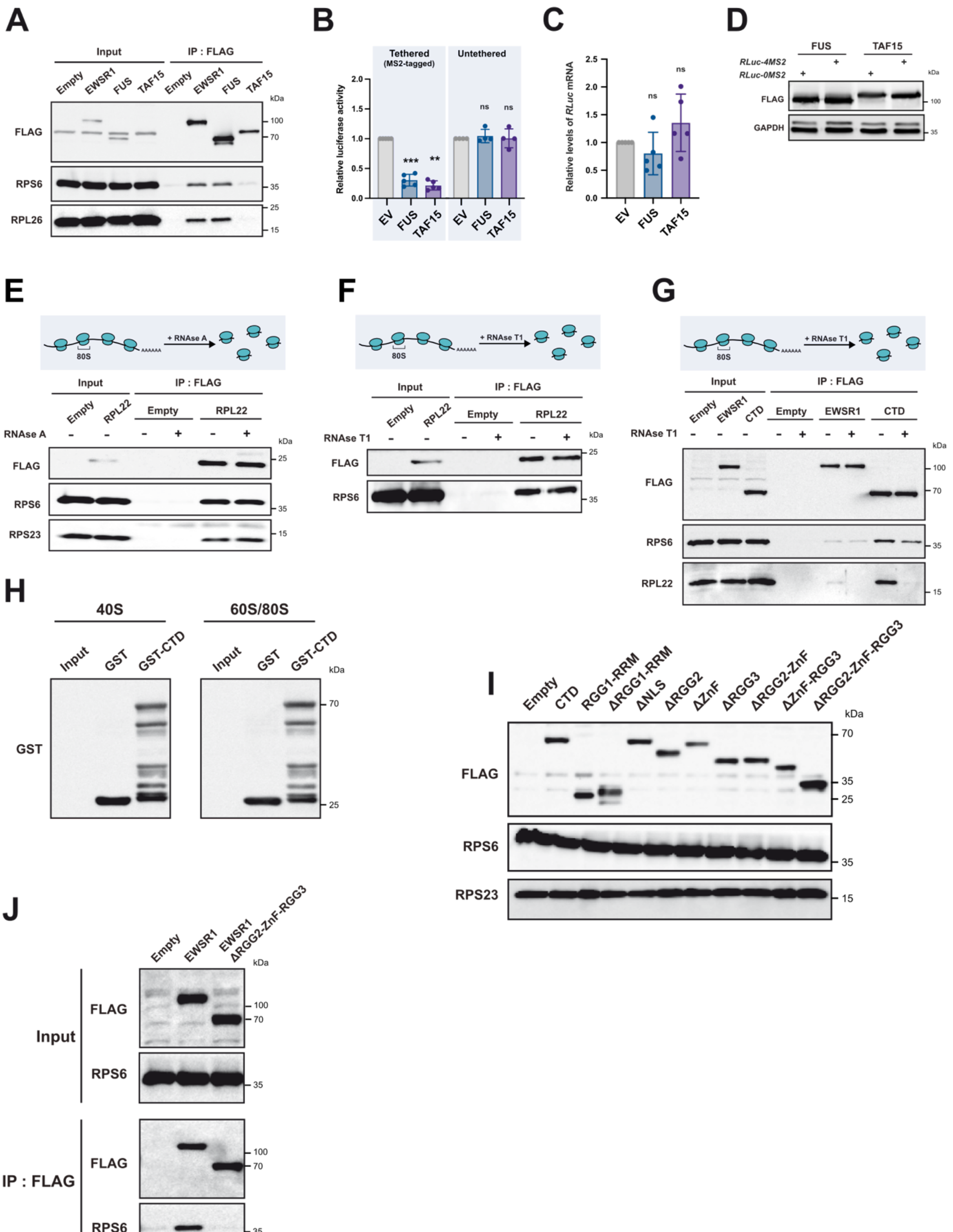

Supplementary Figure 4

Supplementary Figure S4. (**A**) Western blot analysis of FLAG immunoprecipitates (IP) from HeLa cells transfected with FLAG-FUS, FLAG-TAF15 or control FLAG Empty-vector using the indicated antibodies. RPS6 and RPL26 are used as representative components of 40S and 60S, respectively. (**B,C**) Luciferase tethering

assays in HeLa cells transfected with constructs coding for MS2-CP-tagged (tethered) or untagged (untethered) versions of FLAG-EV (EV) or FLAG-tagged FUS or TAF15 together with *RLuc-4MS2* or *RLuc-0MS2* reporters. Results are means of normalized RLuc/FLuc activities [(RLuc-4/FLuc)/(RLuc-0/FLuc)] (**B**) and *RLuc* mRNA levels (**C**), relative to the FLAG-EV condition ± SD from five independent experiments. Statistical analyses were performed on the raw RLuc/FLuc ratios using repeated-measures one-way ANOVA followed by Dunnett's multiple-comparisons test against the FLAG-EV condition. **P < 0.01, ***P<0.001, ns = not significant. (**D**) FLAG western blot analysis of the levels of MS2-FLAG-FUS and MS2-FLAG-TAF15. GAPDH is used as a loading control. Samples are total cell lysates from HeLa cells transfected as described in (**B**). (**E**) (Top) Schematic representation of the effect of RNase A treatment on polysomes. (Bottom) Western blot analysis of FLAG immunoprecipitates (IP) from HeLa cells transfected with FLAG-RPL22 or control FLAG Empty-vector using the indicated antibodies. RPS6 and RPS23 are used as representative components of 40S. Cell lysates were treated (+) or not (-) with RNase A prior to immunoprecipitation. Results show that RPL22 associates with 40S upon treatment with RNase A, indicating that the integrity of monosomes is preserved. (**F**) (Top) Schematic representation of the effect of RNase T1 treatment on polysomes. (Bottom) Western blot analysis of FLAG immunoprecipitates (IP) from HeLa cells transfected with FLAG-RPL22 or control FLAG Empty-vector using the indicated antibodies. RPS6 is used as a representative component of 40S. Cell lysates were treated (+) or not (-) with RNAse T1 prior to immunoprecipitation. Results show that RPL22 associates with 40S upon treatment with RNase T1, indicating that the integrity of monosomes is preserved. (**G**) (Top) Schematic representation of the effect of RNase T1 treatment effect on polysomes. (Bottom) Western blot analysis of FLAG immunoprecipitates (IP) from HeLa cells transfected with FLAG-EWSR1, FLAG-CTD or control FLAG Empty-vector using the indicated antibodies. RPS6 are RPL22 are used as a representative component of 40S and 60S, respectively. Cell lysates were treated (+) or not (-) with RNase T1 prior to immunoprecipitation. (**H**) Western blot analysis of GST-fused proteins in pulldowns performed with GST-CTD or GST alone on 40S or 60S/80S fractions as described in **Fig 4D**. (**I**) Western blot analysis of immunoprecipitation experiments described in **Fig 4E** and showing comparable expression of FLAG-tagged versions of wild-type EWSR1-CTD or the indicated deletion mutants in total lysates after transfection in HeLa cells. RPS6 and RPS23 are used as representative components of 40S and

loading controls. (**J**) Western blot analysis of FLAG immunoprecipitates (IP) from HeLa cells transfected with FLAG-tagged versions of full-length EWSR1, an EWSR1 mutant lacking the RGG2-ZnF-RGG3 region (EWSR1∆RGG2-ZnF-RGG3) or control FLAG Empty-vector using the indicated antibodies. RPS6 is used as a representative component of 40S.

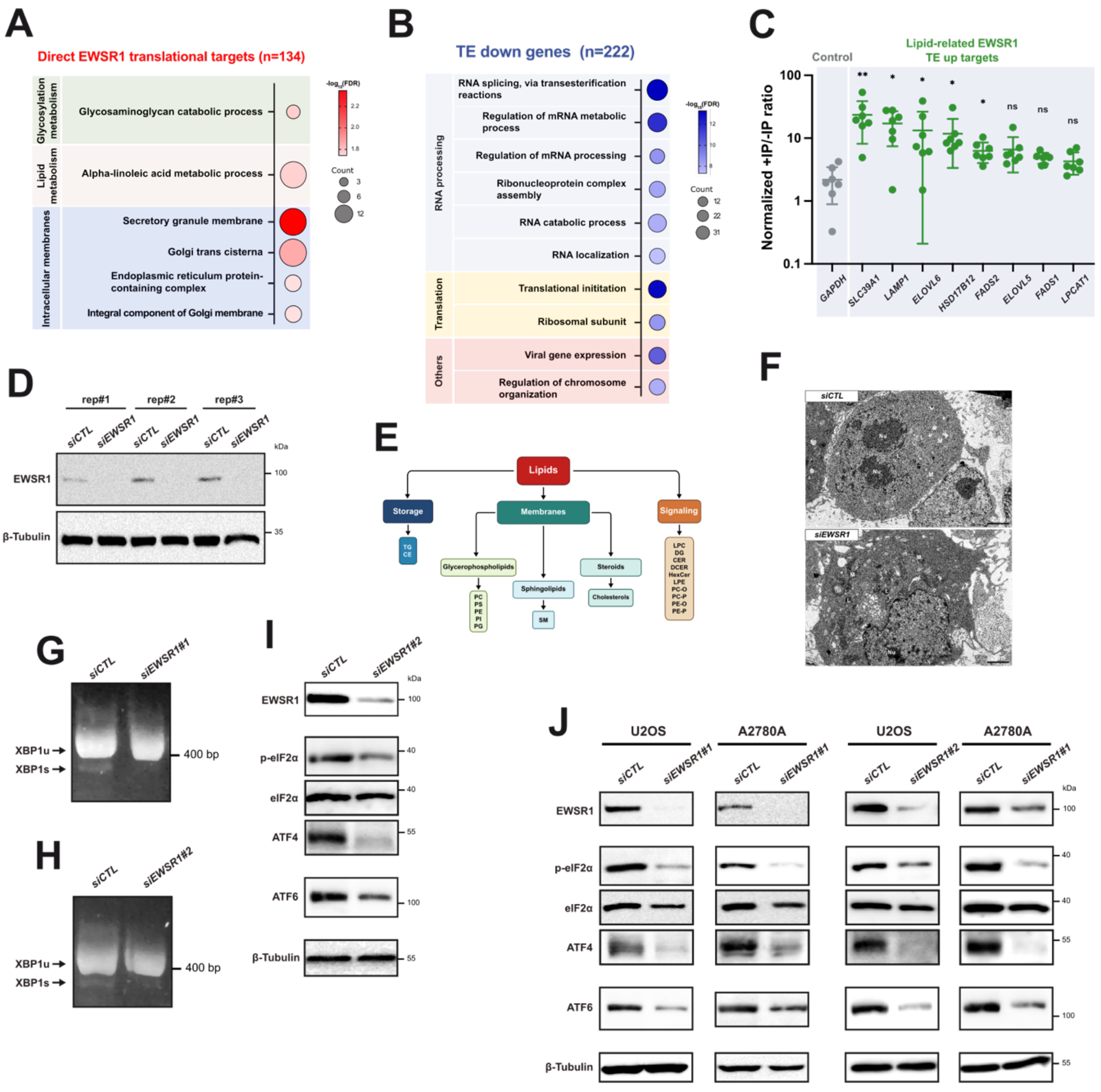

Supplementary Figure S5

Supplementary Figure S5. (**A,B**) Bubble charts of Gene Ontology (GO) Biological Process terms in (**A**) mRNAs identified as "direct EWSR1 translational targets" or (**B**) in TE down mRNAs (blue colored bubbles) in **Fig 2A** (FDR < 0.05). Bubble's size reflects the number of DTEGs and color scale corresponds to P-values, as indicated in the scales. P-values were calculated using FDR correction. (**C**) Association of EWSR1 with the TE up mRNA linked to lipid metabolism as assessed by RIP-qPCR in HeLa cells. Enrichment was calculated as the ratio of specific versus control immunoprecipitation (+IP/-IP) and normalized to 18S rRNA. Data are shown as individual experiments with means ± SD from seven independent experiments. Statistical analyses were performed on log_2-transformed normalized enrichment

values using repeated-measures one-way ANOVA followed by Dunnett's multiple-comparisons test against *GAPDH* mRNA. *P < 0.05, **P < 0.01, ns = not significant. (**D**) Western blot analysis of EWSR1 and β-Tubulin (loading control) in total cell lysates from HeLa cells transfected with *siEWSR1#1* or *siCTL* for 48h and used in **Fig 5B**. (**E**) Lipids classification into three main categories: storage (blue), membrane (green) and signaling (orange). TG: Triacylglyceride, CE: Cholesterol ester, PC: phosphatidylcholine, PS: phosphatidylserine, PE: phosphatidylethanolamine, PI: phosphatidylinositol, PG: phosphatidylglycerol, SM: Sphingomyelin, LPC: lysophosphatidylcholine, DG: diacylglycerides, CER: ceramides, DCER: dihydroceramides, HexCer: hexosylceramides, LPE: lysophosphatidylethanolamine, PC-O: 1-alkyl,2-acylphosphatidylcholine, PC-P: 1- alkenyl,2-acylphosphatidylcholine, PE-O: 1-alkyl,2-acylphosphatidylethanolamine, PE-P: 1-alkenyl,2-acylphosphatidylethanolamine. (**F**) TEM images of HeLa cells transfected with *siCTL* or *siEWSR1#1* for 48h. L: Lysosomes, M: Mitochondria, Nu: Nucleolus. Scale bars: 2 µm. (**G, H**) Agarose gel analysis of the unspliced (u) and spliced (s) XBP1 isoforms amplified by RT-PCR from RNA extracted from HeLa cells transfected for 48h with (**G**) *siCTL* or *siEWSR1#1*, or (**H**) with *siCTL* or *siEWSR1#2*. (**I**) Representative western blot analysis of EWSR1 and components of the PERK and ATF6 branches of the UPR^{ER} in total cell lysates from HeLa cells transfected with *siCTL* or *siEWSR1#2* for 48h. β-Tubulin is used as a loading control. (**J**) Representative western blot analysis of EWSR1 and components of the PERK and ATF6 branches of the UPR^{ER} in total cell lysates from U2OS and A2780A cells transfected with *siCTL*, *siEWSR1#1* or *siEWSR1#2* for 48h. β-Tubulin is used as a loading control.
